# Directionally encoded color track density imaging in brain tumor patients: A potential application to neuro-oncology surgical planning

**DOI:** 10.1016/j.nicl.2023.103412

**Published:** 2023-04-20

**Authors:** Jared J. Sullivan, Leo R. Zekelman, Fan Zhang, Parikshit Juvekar, Erickson F. Torio, Adomas Bunevicius, Walid I. Essayed, Dhiego Bastos, Jianzhong He, Laura Rigolo, Alexandra J. Golby, Lauren J. O'Donnell

**Affiliations:** a Department of Radiology, Brigham and Women’s Hospital, Harvard Medical School, 75 Francis St., Boston, MA 02115, United States; b Department of Neurosurgery, Brigham and Women’s Hospital, Harvard Medical School, 60 Fenwood Rd., Boston, MA 02115, United States

**Keywords:** Track density imaging, Directionally encoded color maps, Brain tumor, Neurosurgical planning

## Abstract

**Background:**

Diffusion magnetic resonance imaging white matter tractography, an increasingly popular preoperative planning modality used for pre-surgical planning in brain tumor patients, is employed with the goal of maximizing tumor resection while sparing postoperative neurological function. Clinical translation of white matter tractography has been limited by several shortcomings of standard diffusion tensor imaging (DTI), including poor modeling of fibers crossing through regions of peritumoral edema and low spatial resolution for typical clinical diffusion MRI (dMRI) sequences. Track density imaging (TDI) is a post-tractography technique that uses the number of tractography streamlines and their long-range continuity to map the white matter connections of the brain with enhanced image resolution relative to the acquired dMRI data, potentially offering improved white matter visualization in patients with brain tumors. The aim of this study was to assess the utility of TDI-based white matter maps in a neurosurgical planning context compared to the current clinical standard of DTI-based white matter maps.

**Methods:**

Fourteen consecutive brain tumor patients from a single institution were retrospectively selected for the study. Each patient underwent 3-Tesla dMRI scanning with 30 gradient directions and a b-value of 1000 s/mm^2^. For each patient, two directionally encoded color (DEC) maps were produced as follows. DTI-based DEC-fractional anisotropy maps (DEC-FA) were generated on the scanner, while DEC-track density images (DEC-TDI) were generated using constrained spherical deconvolution based tractography. The potential clinical utility of each map was assessed by five practicing neurosurgeons, who rated the maps according to four clinical utility statements regarding different clinical aspects of pre-surgical planning. The neurosurgeons rated each map according to their agreement with four clinical utility statements regarding if the map 1 identified clinically relevant tracts, (2) helped establish a goal resection margin, (3) influenced a planned surgical route, and (4) was useful overall. Cumulative link mixed effect modeling and analysis of variance were performed to test the primary effect of map type (DEC-TDI vs. DEC-FA) on rater score. Pairwise comparisons using estimated marginal means were then calculated to determine the magnitude and directionality of differences in rater scores by map type.

**Results:**

A majority of rater responses agreed with the four clinical utility statements, indicating that neurosurgeons found both DEC maps to be useful. Across all four investigated clinical utility statements, the DEC map type significantly influenced rater score. Rater scores were significantly higher for DEC-TDI maps compared to DEC-FA maps. The largest effect size in rater scores in favor of DEC-TDI maps was observed for clinical utility statement 2, which assessed establishing a goal resection margin.

**Conclusion:**

We observed a significant neurosurgeon preference for DEC-TDI maps, indicating their potential utility for neurosurgical planning.

## Highlights

• White matter mapping in brain tumor patients using directionally encoded color (DEC)
• Diffusion tensor vs. advanced DEC maps from post-tractography track density imaging.
• Expert raters prefer advanced DEC maps for neurosurgical planning.
• These maps could help guide neurosurgical planning for brain tumor resections.
• Clinically typical dMRI acquisition parameters allow for generalizability of results.

## 1 Introduction

**Fig. 1:**
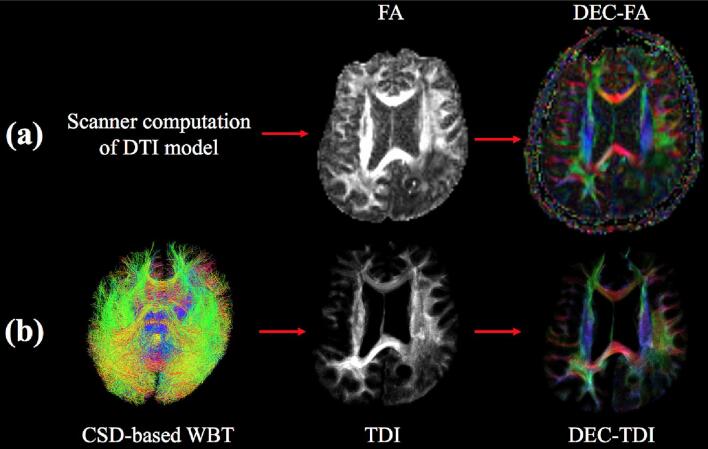
Overview of (a) DEC-FA and (b) DEC-TDI map creation with representative visualizations shown for brain tumor patient 1. CSD = constrained spherical deconvolution, DEC = directionally encoded color, DTI = diffusion tensor imaging, FA = fractional anisotropy, TDI = track density imaging, WBT = whole brain tractography.

Diffusion magnetic resonance imaging (dMRI) detects the naturally occurring diffusion of water in the body as a means to infer the underlying tissue structure (Le Bihan and Johansen-Berg, 2012). In oncological brain surgery, the surgeon must balance two important factors: maximizing resection of the tumor and minimizing damage of surrounding critical brain tissue (Essayed et al., 2017). Through preoperative dMRI imaging and tractography, neurosurgeons can identify the location of the tumor relative to eloquent white matter tracts that must be preserved (Becker et al., 2020, Essayed et al., 2017, Henderson et al., 2020, Panesar et al., 2019, Yang et al., 2021). dMRI is often visualized clinically using directionally encoded color (DEC) maps (Pajevic and Pierpaoli, 2000) (Fig. 1). DEC maps employ a standardized color scheme (blue = superior-inferior, red = left–right, green = anterior-posterior) to represent the orientation of the white matter fiber tracts being visualized. Preoperative DEC map visualization may aid in assessing the type and degree of white matter tract involvement in brain tumor patients including whether tracts have been infiltrated, displaced or destroyed by the tumor (Field et al., 2005, Field et al., 2004, Schneider et al., 2021, Schonberg et al., 2006, Witwer et al., 2002, Young, 2007).

Traditional DEC maps are based on the diffusion tensor imaging (DTI) model, where the major eigenvector of the tensor model represents the primary white matter fiber orientation of the tissue (Basser et al., 1994). In these traditional DEC maps, the brightness of each voxel reflects the local fractional anisotropy (FA), a measure of the variation in diffusion direction (O’Donnell and Westin, 2011). For clarity, in the rest of this report we will refer to these traditional DEC maps as DEC-FA maps.^1^ Because DEC-FA maps are automatically produced by the MRI scanner from clinical DTI sequences, they provide a convenient and rapid method for visualizing white matter tracts.

However, the DTI model, which only represents a single tract orientation per voxel, has a limited capability to resolve areas of crossing fiber tracts (Farquharson et al., 2013, Jeurissen et al., 2013). Another well described limitation of DTI is its less robust modeling of fiber orientation in regions containing peritumoral edema in the setting of brain lesions (Essayed et al., 2017, Gong et al., 2018, Zhang et al., 2013). Finally, the spatial resolution of clinical DTI may be insufficient to enable visualization of fine details of white matter structure (Calamante et al., 2010). Higher-order multi-fiber models, such as constrained spherical deconvolution (CSD) (Tournier et al., 2007), potentially overcome many limitations of DTI-based methods, including the modeling of crossing fibers and improved tracking through edema (Becker et al., 2020, Chen et al., 2015, Essayed et al., 2017, Gong et al., 2018, Henderson et al., 2020, Mormina et al., 2015).

Track Density Imaging (TDI) is a technique that leverages dMRI tractography to create a white matter map with enhanced resolution relative to the acquired dMRI data (Calamante et al., 2013, Calamante et al., 2010). TDI maps enable high-resolution visualization of the track density, defined as the number of tractography streamlines passing through each image voxel. It has been suggested that high-resolution TDI maps may confer additional anatomical detail to neurosurgeons planning brain tumor resections (Calamante et al., 2010, Wei et al., 2017). The TDI framework can be used to create high-resolution DEC maps, where color represents the average direction of all tractography streamlines passing through a given voxel, and brightness is determined by the local track density (Calamante et al., 2010). We refer to these maps as DEC-TDI (Fig. 1). These maps benefit from white matter tractography based on a higher-order multi-fiber model, which has known advantages for neurosurgical planning (Becker et al., 2020, Essayed et al., 2017, Farquharson et al., 2013, Henderson et al., 2020, Mormina et al., 2015). Therefore, DEC-TDI may offer improved white matter visualization in patients with brain tumors.

The main aim of this study was to assess the potential of DEC-TDI as a tool for neuro-oncology surgical planning. We compared the potential clinical utility of DEC-TDI maps versus scanner default DEC-FA maps according to neurosurgeons’ expert rating. We developed four clinical utility statements to rate the maps. Five practicing neurosurgeons participated in the expert rating process. We found rater scores were significantly higher for DEC-TDI maps than DEC-FA maps on data from fourteen consecutive brain tumor patients. Overall, we observed a general neurosurgeon preference for DEC-TDI maps, indicating their potential utility for neurosurgical planning.

## 2 Methods

### 2.1 Subjects

Sixteen consecutive brain tumor patients treated by a single neurosurgeon [AJG] with clinical MRI scans collected from 8/22/2019–7/28/2020 at Brigham and Women’s Hospital in Boston, MA were identified. One patient was excluded due to excessive motion artifacts and another was excluded due to a processing error in the tumor segmentation map, leaving fourteen (eight male, six female; average age, 55.6 years, age range 23–73 years) for inclusion in this study (Table 1). Tumor grade breakdown of the included study sample was: one grade II, one grade III, seven grade IV, one unclear grade, and four metastatic disease. Handedness was determined according to the Edinburgh Handedness Inventory for all patients except for BTP 2, whose handedness is self-reported. The study was approved by the Partners Healthcare Institutional Review Board, and written informed consent was obtained from all subjects prior to participation.

**Table 1:** Patient characteristics. Peritumoral edema is categorized as extensive (spanning > 50% of a single lobe or multiple lobes), limited (spanning < 50% of a single lobe), or none. BTP = brain tumor patient, GBM = glioblastoma multiforme, WHO = World Health Organization.

Patient	Sex	Age (y)	Handedness	Pathology Diagnosis	WHO Grade	Tumor Location	Tumor diameter (mm)	Peritumoral edema (none, limited, extensive)
BTP 1	M	73	Right	Metastasis	N/A	Left parietal	22.3	Extensive
BTP 2	M	69	Right	Metastasis	N/A	Left frontal	11.9	Extensive
BTP 3	M	68	Left	Oligodendro-glioma	III	Left frontal	49.1	Limited
BTP 4	F	39	Right	GBM	IV	Left temporal	14.4	Limited
BTP 5	F	23	Right	GBM	IV	Left frontal	30.8	Extensive
BTP 6	M	53	Right	GBM	IV	Left temporal	44.2	Limited
BTP 7	M	63	Right	GBM	IV	Left frontal	18.0	Extensive
BTP 8	M	47	Right	Astrocytoma	Unclear	Right temporal	62.9	None
BTP 9	M	67	Left	GBM	IV	Right frontal	24.7	Limited
BTP 10	F	65	Right	Metastasis	N/A	Left frontal	17.8	Limited
BTP 11	F	66	Right	GBM	IV	Right frontal	11.0	Limited
BTP 12	F	32	Right	Astrocytoma	II	Left temporal	38.5	None
BTP 13	M	53	Ambidex- trous	GBM	IV	Right temporal	69.6	Extensive
BTP 14	F	53	Ambidex- trous	Metastasis	N/A	Left frontal	54.3	Extensive

### 2.2 Data acquisition and DEC-FA map generation

Preoperative structural and diffusion images were acquired using Siemens 3T scanners (Siemens Trio and Verio, Siemens Healthcare, Erlangen, Germany). Diffusion-weighted images (DWI) were acquired using an echo planar imaging (EPI) sequence (30 gradient directions, 6 baseline (b = 0) images, b = 1000 s/mm^2^, TR = 3200 ms, TE = 58 ms, flip angle = 90°, voxel size = 2.0 mm isotropic). DEC-FA DICOM files were output directly by the MRI scanner and were computed from the DWI data using diffusion tensor modeling. T1-weighted scans (TR = 1900–2000 ms, TE = 252–340 ms, flip angle = 9–15°, voxel size = 1.0 mm isotropic) and T2-weighted scans (TR = 2000 ms, TE = 232 ms, flip angle = 120°, voxel size = 1.0 mm isotropic) were acquired as clinically indicated for each patient.

### 2.3 DWI data preprocessing

We applied a minimal preprocessing pipeline that has been applied in several of our clinical studies (Chen et al., 2016, Chen et al., 2015, Gong et al., 2018, O’Donnell et al., 2017). DEC-FA and DWI DICOM data were converted to NIFTI format using the *Diffusion-weighted DICOM Import (DCM2niixGUI)* module (Li et al., 2016) in 3D Slicer (https://www.slicer.org) (Fedorov et al., 2012) via the SlicerDMRI project (https://dmri.slicer.org) (Norton et al., 2017, Zhang et al., 2020b). Binary brain masks were automatically generated in 3D Slicer using the *Diffusion Brain Masking* module. MP-PCA denoising was applied to the NIFTI DWI data using the *MRTrix3* software package (J-D Tournier, Brain Research Institute, Melbourne, Australia; https://github.com/MRtrix3/mrtrix3) (Tournier et al., 2019, Veraart et al., 2016). This step is recommended to reduce the contribution of noise to anatomical details seen in TDI maps (Dhollander et al., 2014, Dhollander et al., 2012). We applied DTIPrep (https://www.nitrc.org/projects/dtiprep) (Oguz et al., 2014) to perform motion and eddy current distortion correction of the denoised DWI data.

### 2.4 Tractography

Whole brain single tissue probabilistic constrained spherical deconvolution (CSD)-based tractography without anatomical constraints was performed for each patient DWI dataset in *MRTrix* using the second order integration over fiber orientation distributions (iFOD2) algorithm (Tournier et al., 2007, Tournier et al., 2010). Tractography streamlines were randomly seeded within the binary brain masks generated for each patient. Tractography parameters were chosen to be consistent with previous TDI studies (Calamante et al., 2010, Calamante et al., 2011, Calamante et al., 2012), as follows. We used a sufficiently large number of streamlines (2 million) to achieve a nearly continuous white matter representation for TDI (Calamante et al., 2010, Calamante et al., 2011). The tractography step size (0.3 mm) was set smaller than the TDI grid size (0.5 mm) to enable TDI to exploit subvoxel tractography information (Calamante et al., 2010). The maximum harmonic order was set to 6 based on the number of DWI gradient directions (Tournier et al., 2009). The remaining tractography parameters were set to default values for consistency with TDI studies (Calamante et al., 2010, Calamante et al., 2011, Calamante et al., 2012) and recent CSD-based glioma patient tractography studies (Fekonja et al., 2020, Schult et al., 2019, Sheng et al., 2021), as follows: maximum branching angle = 45°, 4 FOD samples per step, minimum fiber length = 10 mm (5 × voxel size), maximum fiber length = 200 mm (100 × voxel size), fiber orientation distribution (FOD) amplitude threshold for seeding/stopping = 0.1.

### 2.5 DEC-TDI map generation

CSD-derived DEC-TDI maps were calculated in *MRtrix* at 0.5 mm isotropic grid size using each patient’s baseline b = 0 image as a template for consistent track display in diffusion space (Calamante et al., 2010). The grid size determines the image resolution of the DEC-TDI map, and a grid size of 0.5 mm is consistent with the TDI literature (Barajas et al., 2013, Calamante et al., 2010, Dhollander et al., 2014, Woodworth et al., 2015, Ziegler et al., 2014).

### 2.6 Tumor segmentation and creation of visualizations for expert rating

**Fig. 2:**
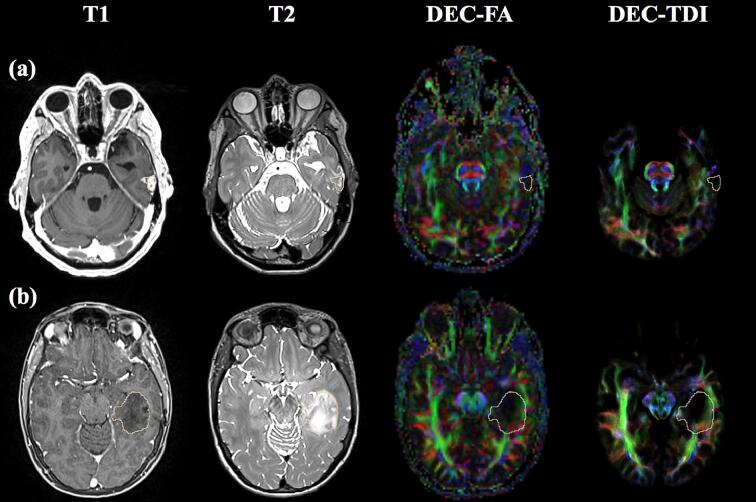
Example visualizations from two patients. (a) BTP 4, 39-year old female with glioblastoma, (b) BTP 12, 32-year old female with astrocytoma. Shown from left to right: single axial slices of the T1-weighted image, T2-weighted image, DEC-FA map, and DEC-TDI map. Tumor outlines are provided in pink on each image for reference. DEC = directionally encoded color, FA = fractional anisotropy, T1 = T1-weighted image, T2 = T2-weighted image, TDI = track density imaging. (For interpretation of the references to color in this figure legend, the reader is referred to the web version of this article.)

For visualization, each patient’s T1- and T2-weighted images, DEC-FA and DEC-TDI maps were rigidly aligned using the *General Registration (BRAINS)* and *Transforms* modules in 3D Slicer. A physician with significant surgical planning experience [PJ] segmented brain tumors in 3D Slicer with reference to preoperative T1- and/or T2-weighted brain MR images with and without gadolinium contrast. Tumor diameters were then measured in axial slices using the *Annotations* module in 3D Slicer. For each patient, the single axial slice with the largest tumor diameter was selected for visualization for expert rating. If a patient had multiple lesions, the diameters of the individual lesions were summed to determine the appropriate axial slice. Then screenshots of the selected axial slices were captured (Fig. 2) including T1- and T2- weighted images, DEC-FA, and DEC-TDI maps. For anatomical reference, tumor outlines were overlaid on all images. For consistent comparison across DEC maps, the brightness and contrast was tuned to provide a similar image intensity: DEC-FA maps were viewed at a window level of 100 and window width of 200, while DEC-TDI maps were viewed at a window level of 75 and a window width of 150.

### 2.7 Clinical utility criteria

We defined criteria for an effective pre-surgical planning white matter visualization with the input of two practicing neurosurgeons [PJ, AG]. The neurosurgeons recommended assessing the ability of each map to help demonstrate clinically relevant peritumoral white matter tracts, to establish a goal margin of resection, and to influence the planned surgical route, as well as to assess the overall usefulness of each map. Therefore, we developed the following four clinical utility statements for evaluation of DEC maps:

1. This DEC map demonstrates clinically relevant tracts in the peritumoral region (i.e. areas of T1/T2 signal change).
2. The resolution of this DEC map is helpful in establishing a goal margin of resection.
3. This DEC map would influence my planned surgical route.
4. Overall, this DEC map is useful.

### 2.8 Expert rating

Five MDs [EFT, AB, PJ, WIE, DB] with extensive neurosurgical training performed expert rating to assess how well each DEC map met the four clinical utility criteria described above. To ensure that raters were blinded to map type and patient, the 28 DEC maps (two per patient) were presented in a fully randomized order. Rating was performed using a survey in Qualtrics (Qualtrics, Provo, UT) following similar past methodology for expert rating of visualizations (Franke et al., 2021, Tie et al., 2014). The survey displayed images of the selected axial image slice (as described in Section 2.6) of a DEC-FA or DEC-TDI map, alongside the corresponding T1-weighted and T2-weighted images. (A visualization of all patient DEC maps as presented to the raters is provided in Supplementary Fig. 1.) After viewing each DEC map, the expert raters were asked to rate the degree to which they agreed with the four clinical utility statements described in Section 2.7. The expert responses followed a 5-point Likert scale (Likert, 1932): strongly disagree (1), somewhat disagree (2), neither agree nor disagree (3), somewhat agree (4), strongly agree (5). Each expert response was therefore recorded as a “rater score” between 1 and 5.

### 2.9 Statistical analysis

Our primary goal was to compare the DEC map types (DEC-FA or DEC-TDI) by assessing how map type impacted rater score for each clinical utility statement. The data were modeled using a two-way repeated ordinal regression with a cumulative link mixed effect model (CLMM) (McCullagh, 1980) following the methodology detailed by Mangiafico (2016). This model was chosen for its simplicity and efficacy in evaluating the individual effects of several variables (i.e., expert rater, map type, and interaction between expert rater and map type) on an ordinal dependent variable (i.e. rater scores via a 5-point Likert scale) (McCullagh, 1980). To account for individual differences across patients, a patient blocking variable was added to the models; likelihood ratio tests (Satorra and Saris, 1985) were conducted to assess the goodness of fit of its inclusion. The hypothesis that map type influenced rater score was tested using a type II analysis of deviance (ANODE) based on Wald χ^2^ tests (Christensen, 2018, Scheffé, 1999). In a post-hoc analysis, the effect of map type (DEC-FA vs. DEC-TDI) on rater score was measured using an estimated marginal means (EMM) analysis (Lenth and Love, 2018). Significance thresholds were set at α = 0.05. Analyses were performed in R, a statistical software program (R Core Team, 2020), using the “clmm,” “car,” “emmeans,” and “ggplot2” packages (Christensen, 2018, Kahle and Wickham, 2013, Lenth and Love, 2018).

## 3 Results

### 3.1 Data acquisition time

DWI data acquisition time was approximately 2–3 min per subject. Average tractography runtime across included subjects was 81.1 ± 12.1 min. DEC-TDI maps were generated in less than one minute following tractography.

### 3.2 Summary of rater responses

**Fig. 3:**
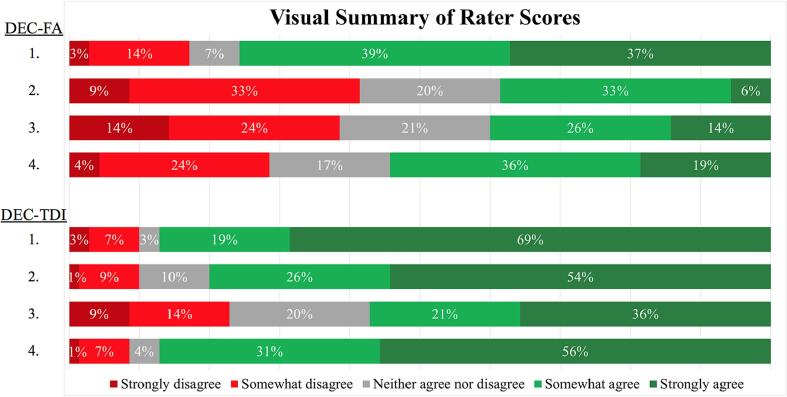
Visual summary of rater scores. Stacked bar chart visualization of overall rater score results separated by map type (top: DEC-FA, bottom: DEC-TDI) for each of the four clinical utility statements (1. Identifies clinically relevant tracts, 2. Helps establish goal resection margin, 3. Influences planned surgical route, 4. Overall, is useful). Bars are labeled with the percentage of ratings receiving each score. DEC = directionally encoded color, FA = fractional anisotropy, TDI = track density imaging.

Fig. 3 shows aggregate rater score data (across the five neurosurgeons and fourteen patients). Expert raters found both maps to be useful overall: it can be observed that a majority of rater responses are shown in green (indicating “somewhat agree” or “strongly agree”) for both map types. Furthermore, based on the larger number of green responses for DEC-TDI, it is apparent that there is a general preference among the raters for the DEC-TDI maps (see Section 3.4 for statistical analyses).

### 3.3 Visualization of selected patient data

**Fig. 4:**
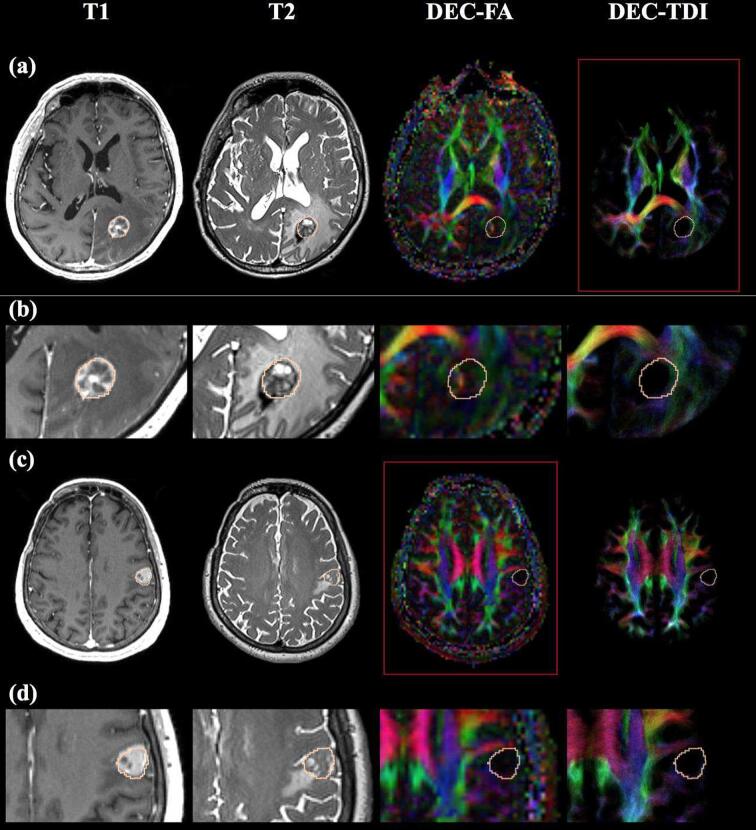
DEC maps illustrating expert rater preferences. DEC maps from BTP 1 ((a) full axial view, (b) zoomed-in peritumoral view), whose DEC-TDI map was consistently preferred over the DEC-FA map. DEC maps from BTP 10 ((c) full axial view, (d) zoomed-in peritumoral view), whose DEC-FA map was slightly preferred over the DEC-TDI map. Red boxes in rows (a) and (c) indicate the preferred map for each patient. Shown from left to right: single axial slices of the T1-weighted image, T2-weighted image, DEC-FA map, and DEC-TDI map. Tumor outlines are provided in pink on each image for reference. DEC = directionally encoded color, FA = fractional anisotropy, T1 = T1-weighted image, T2 = T2-weighted image, TDI = track density imaging. (For interpretation of the references to color in this figure legend, the reader is referred to the web version of this article.)

Example cases were selected to illustrate neurosurgeon preferences for a particular DEC map. Fig. 4 shows one example patient (BTP 1) where the DEC-TDI map was preferred over the DEC-FA map with the largest difference in raw rater scores. The largest rater score difference in favor of DEC-TDI was observed for clinical utility statement 2, “The resolution of this DEC map is helpful in establishing a goal margin of resection.” This indicates that visualization of the tumor margin was the most important reason raters preferred this DEC-TDI map. Fig. 4 also shows the only patient (BTP 10) where the DEC-FA map was generally preferred over the DEC-TDI map. The largest rater score difference in favor of DEC-FA was observed for clinical utility statement 1, “This DEC map demonstrates clinically relevant tracts in the peritumoral region (i.e. areas of T1/T2 signal change).” This indicates that in this case, the visualization of apparent fiber tracts was a driving factor behind the rater preference for the DEC-FA map.

### 3.4 Statistical analyses

For all four clinical utility statements, the cumulative model including patient as a random blocking variable significantly outperformed the model not including a blocking variable (Supplementary Table 1). Therefore, the cumulative model including the blocking variable, patient, was used for the following analyses.

The analysis of deviance results for the variables included in the cumulative model are reported in Table 2. Across the four clinical utility statements, the primary explanatory variable, map type, had a statistically significant effect on rater score. This finding indicates that map type significantly influenced rater score across all four investigated clinical utility statements. Both secondary explanatory variables, rater and map/rater interaction, also reached statistical significance across all clinical utility statements, indicating that rater and the interaction between map type and rater also influenced rater score.

**Table 2:** Analysis of deviance results. A significant result indicates a significant contribution of a given explanatory variable on rater score. Map type was the primary explanatory variable of interest. χ^2^ = chi-squared statistic, df = degrees of freedom, N = number of data points for each statistical test.

Clinical Utility Statements	Explanatory Variables					
	Map type		Rater		Map type/Rater Interaction	
	χ^2^ (df = 1, N = 140)	p-value	χ^2^ (df = 4, N = 140)	p-value	χ^2^ (df = 4, N = 140)	p-value
1. Identifies clinically relevant tracts	12.9	0.00033	12.6	0.013	23.5	0.00010
2. Helps establish goal resection margin	55.5	<0.0001	26.6	<0.0001	41.8	<0.0001
3. Influences planned surgical route	10.6	0.0011	11.8	0.019	17.7	0.0014
4. Overall, is useful	38.6	<0.0001	29.3	<0.0001	18.6	0.00094

**Fig. 5:**
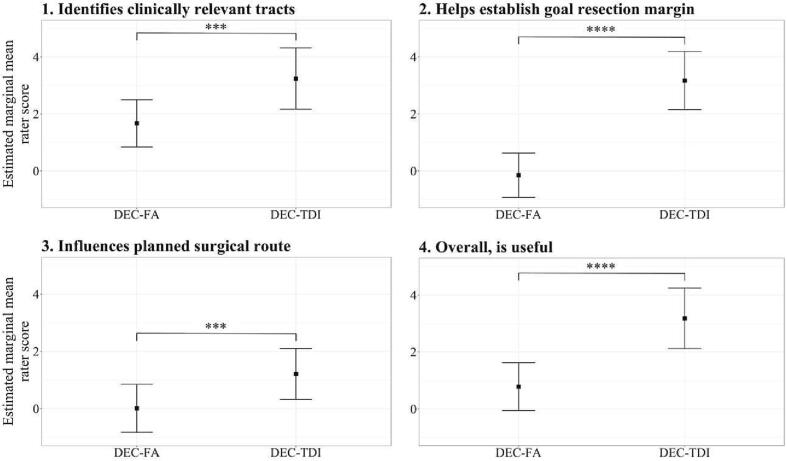
Plot of estimated marginal mean (EMM) rater scores for the four clinical utility statements. Error bars indicate the 95% confidence interval of the EMM. Significant differences between map types are indicated with stars (*** = p ≤ 0.001, **** = p ≤ 0.0001). DEC = directionally encoded color, FA = fractional anisotropy, TDI = track density imaging.

The results of the estimated marginal means (EMM) analysis are plotted in Fig. 5. The EMM contrast (defined as the difference between the estimated marginal mean rater score for DEC-TDI and DEC-FA maps) was statistically significant in all analyses, showing a general expert preference for DEC-TDI maps across all clinical utility statements.

## 4 Discussion

In this study, we have investigated the potential application of traditional and advanced DEC maps to aid neurosurgical planning in brain tumor patients. To our knowledge, this study is the first to specifically assess the utility of DEC-TDI for neurosurgical planning in brain tumor patients. We found that five neurosurgeon expert raters broadly preferred DEC-TDI maps over traditional DTI-based DEC-FA maps in a cohort of 14 brain tumor patients. This study provides evidence that DEC-TDI maps may help neurosurgeons identify important peritumoral tracts, decide on a goal resection margin, and plan a surgical route. To assess why DEC-TDI maps were generally preferred by neurosurgeon raters, we consider the interpretation of clinical utility statements 1 and 2, which assessed specific components of neurosurgical planning. The largest effect size in rater scores in favor of DEC-TDI maps was observed for statement 2, “The resolution of this DEC map is helpful in establishing a goal margin of resection” (Fig. 5). This result suggests that the increased image resolution achievable with DEC-TDI (Calamante et al., 2010) has potential clinical utility to improve visualization of the tumor margin and that DEC-TDI maps could potentially assist neurosurgeons as they establish a planned resection margin. This potential role of DEC-TDI aligns with well-established clinical guidelines that affirm the importance of a precise resection margin that both removes tumor while sparing healthy tissue for patient morbidity and mortality (Nabors et al., 2020) and the potentially impactful role of white matter visualization techniques in determining a resection margin (Dimou et al., 2013, Romano et al., 2009). Rater scores also strongly favored DEC-TDI maps for clinical utility statement 1, “This DEC map demonstrates clinically relevant tracts in the peritumoral region (i.e. areas of T1/T2 signal change)” (Fig. 5). Visualization and identification of white matter tracts and their relationship to the brain tumor is a well-recognized goal of white matter visualization methods for use in presurgical planning (Essayed et al., 2017, Yeh et al., 2021), further supporting the potential role of DEC-TDI maps in this clinical context. Finally, rater scores for clinical utility statements 3 and 4, which were designed to judge the overall influence of each DEC map on a planned surgical route and the overall utility of each DEC map, also significantly favored DEC-TDI, supporting its overall potential to help neurosurgeons plan function-preserving brain tumor resections in conjunction with currently employed imaging techniques. While DEC-TDI was preferred, raters found both DEC maps to be useful, with a majority of “somewhat agree” or “strongly agree” responses for both map types. This result is in agreement with existing studies supporting the utility of preoperative DEC-FA visualization in patients (Field et al., 2005, Field et al., 2004, Schneider et al., 2021, Schonberg et al., 2006, Witwer et al., 2002, Young, 2007). Our findings may generalize to a wide patient population across institutions, given the diversity of brain tumor diagnoses and grades included in the study as well as the clinically typical dMRI data used.

## 5 Limitations & future directions

Our study has several limitations. To make expert rating feasible for neurosurgeons, the study design restricted visualization to a single axial slice of each DEC map. However, in clinical practice, access to the full DEC map could allow for improved tract visualization in relation to the tumor, including in different orientations. A second limitation to the study design is that the DEC-TDI maps enabled visualization only within the brain mask (where tractography was performed), while the DEC-FA maps included all anatomy that was scanned, such as the brain, cerebrospinal fluid, and skull. Our goal was to preserve the default nature of the DEC-FA maps, presenting them to expert raters as they are produced by the scanner without additional processing. The corresponding difference in visual presentation of the maps may have biased raters toward the brain extracted DEC-TDI maps, while providing raters with a way to to identify the map type despite the random, blind presentation of maps. In terms of patient selection, we selected a cohort of consecutive patients for this study with the goal of studying multiple different tumor histopathologic diagnoses, locations, sizes, and degree of edema without any a priori judgment of which cases would be most suited for DEC visualization. A number of patient- and tumor-specific factors therefore impact the potential clinical utility of DEC maps. First, for tumors located near the cortical surface (e.g. BTP 2, 3, 4, 7, 10, and 13), there is relatively less contrast visible in the DEC maps. Similarly, diffusion anisotropy is lower in areas of peritumoral edema, which leads to reduced brightness and contrast in DEC-FA maps and can limit fiber tracking (Essayed et al., 2017, Gong et al., 2018, Zhang et al., 2013), therefore reducing DEC-TDI contrast. Furthermore, DEC-TDI may be most useful in cases where tractography is particularly useful. For these reasons, DEC maps may offer minimal added value to the preoperative plan in some cases. In this work, we performed limited dMRI preprocessing in line with clinical practice and to reduce potential differences between the input data to DEC-TDI and that used for DEC-FA on the scanner. We performed MP-PCA denoising to reduce the known sensitivity of TDI-based visualization to noise (Dhollander et al., 2014, Dhollander et al., 2012), but future work may improve results of DEC-TDI by employing additional DWI preprocessing steps such as Gibbs-ringing correction, EPI distortion correction, and bias field inhomogeneity correction. In addition, all patients received diffusion MRI scanning as clinically indicated. Patient selection was not determined by the lateralization or localization of specific behavioral, cognitive, or neurological functions or clinical functional presentation. Therefore, the importance of DEC visualization in surgical planning with regard to specific behavioral, cognitive, or neurological functions (such as language function) was not investigated in this study and is of interest for future work. Furthermore, we acknowledge that our overall goal is a clinically motivated comparison of a newer method of white matter visualization (DEC-TDI) versus a traditional method (DEC-FA), rather than a technical comparison of these methods. More research is needed to assess which underlying technical factors (e.g. DTI vs CSD modeling, the usage of tractography to produce the DEC-TDI map, and differences in pre-processing on and off the scanner) may be driving expert preferences. The comparison of other types of DEC map, such as that derived from an orientation distribution function (ODF) field to create a directionally encoded color fiber orientation distribution map (DEC-FOD) (Dhollander et al., 2015), is of interest in the future. Finally, this initial study focused on evaluation of retrospective patient DEC maps. In order to move beyond the hypothetical setting of this study to clinical practice, further prospective study is needed to assess the clinical outcomes of brain tumor patients whose preoperative plan includes DEC-TDI.

Other study limitations relate to challenges of clinical implementation of DEC-TDI. While DEC-TDI benefits from the advantages of tractography, it also suffers from limitations of tractography including false positive streamlines and challenges tracing through peritumoral edema that can lead to false negatives (Essayed et al., 2017, Henderson et al., 2020, Yang et al., 2021), as well as potentially cumbersome computational run time. Furthermore, the clinically standard data employed in this project is not ideally suited for CSD modeling, which benefits from data with b-values of 3000 s/mm^2^ and over 45 gradient directions to better model crossing fibers while reducing the effect of noise. DEC-TDI maps derived from tractography that suffers from false positives or false negatives could increase the likelihood of a suboptimal surgical approach. While the DEC-FA maps were automatically generated on the scanner, in our study the average time to generate a DEC-TDI map (including tractography and map generation) was over an hour per patient on a modern Linux computer workstation. In addition, while our study employed recommended and default parameters for tractography and DEC-TDI generation, further study may be needed to optimize and standardize these parameters for generalization across different brain tumor patients and dMRI sequences.

In future clinical applications, DEC-TDI visualization may be useful to aid in virtual dissection and visualization of specific white matter tracts adjacent to the tumor. In conjunction with automated tractography segmentation techniques described previously by our group (O’Donnell et al., 2017, Zhang et al., 2020a), clinically relevant tracts can be clearly visualized and anatomically defined, providing neurosurgeons with an improved presurgical visualization of the important peritumoral white matter tracts that will likely influence the resection strategy.

## 6 Conclusion

Our study demonstrates that preoperative planning white matter visualization using DEC-TDI maps is preferred by expert neurosurgeon raters over the current clinical standard, DEC-FA maps. DEC-TDI may have a potential role in conjunction with currently employed imaging techniques to help neurosurgeons plan function-preserving brain tumor resections. Further study is needed for methodology validation and clinical translation.

## CRediT authorship contribution statement

**Jared J. Sullivan:** Conceptualization, Investigation, Formal analysis, Visualization, Writing – original draft. **Leo R. Zekelman:** Formal analysis, Writing – original draft. **Fan Zhang:** Methodology, Investigation, Writing – review & editing. **Parikshit Juvekar:** Visualization, Investigation. **Erickson F. Torio:** Investigation. **Adomas Bunevicius:** Investigation. **Walid I. Essayed:** Investigation. **Dhiego Bastos:** Investigation. **Jianzhong He:** Data curation. **Laura Rigolo:** . **Alexandra J. Golby:** Supervision, Conceptualization, Investigation, Writing – review & editing. **Lauren J. O'Donnell:** Supervision, Conceptualization, Investigation, Writing – review & editing.

## Declaration of Competing Interest

The authors declare that they have no known competing financial interests or personal relationships that could have appeared to influence the work reported in this paper.

## Data Availability

The data that has been used is confidential.

## Footnotes

1 We note that DEC-FA maps are also referred to as FA color maps (Young, 2007), color by orientation maps (Norton et al., 2017), color-coded maps (Schneider et al., 2021), color-coded FA maps (Voltoline and Wu, 2021), and other similar names.
