# Supplementary material for: Directionally encoded color track density imaging in brain tumor patients: A potential application to neuro-oncology surgical planning
Source: Neuroimage Clin. 2023 Apr 20;38:103412. doi: 10.1016/j.nicl.2023.103412 (PMC10165166; doi:10.1016/j.nicl.2023.103412)
Supplement: Supplementary data 1 [file mmc1.docx]

**Supplementary Materials**

| **Clinical Utility Statement** | **AIC score** | **Log-likelihood** | **P-value** |
| --- | --- | --- | --- |
| 1. Identifies clinically relevant tracts | Model 1: 330.6  Model 2: 320.9 | Model 1: -154.3  Model 2: -148.4 | p = 0.0060 |
| 2. Helps establish goal resection margin | Model 1: 344.9  Model 2: 333.9 | Model 1: -161.4  Model 2: -155.0 | p = 0.00032 |
| 3. Influences planned surgical route | Model 1: 436.5  Model 2: 414.6 | Model 1: -207.2  Model 2: -195.3 | p < 0.0001 |
| 4. Overall, is useful | Model 1: 356.4  Model 2: 340.7 | Model 1: -167.2  Model 2: -158.4 | p < 0.0001 |

**Supplementary Table 1.** Likelihood ratio test results. Model 1: Rater score as a function of map type, expert rater, and the interaction of the two. Model 2: Model 1 + a “patient” blocking variable. p-values are reported for each clinical utility statement for each likelihood ratio test with a significance threshold of α = 0.05. A significant result, along with lower AIC score and higher log-likelihood value indicates better performance of that model compared to the other. AIC = Akaike information criterion.

*BTP 1 DEC-FA*

**
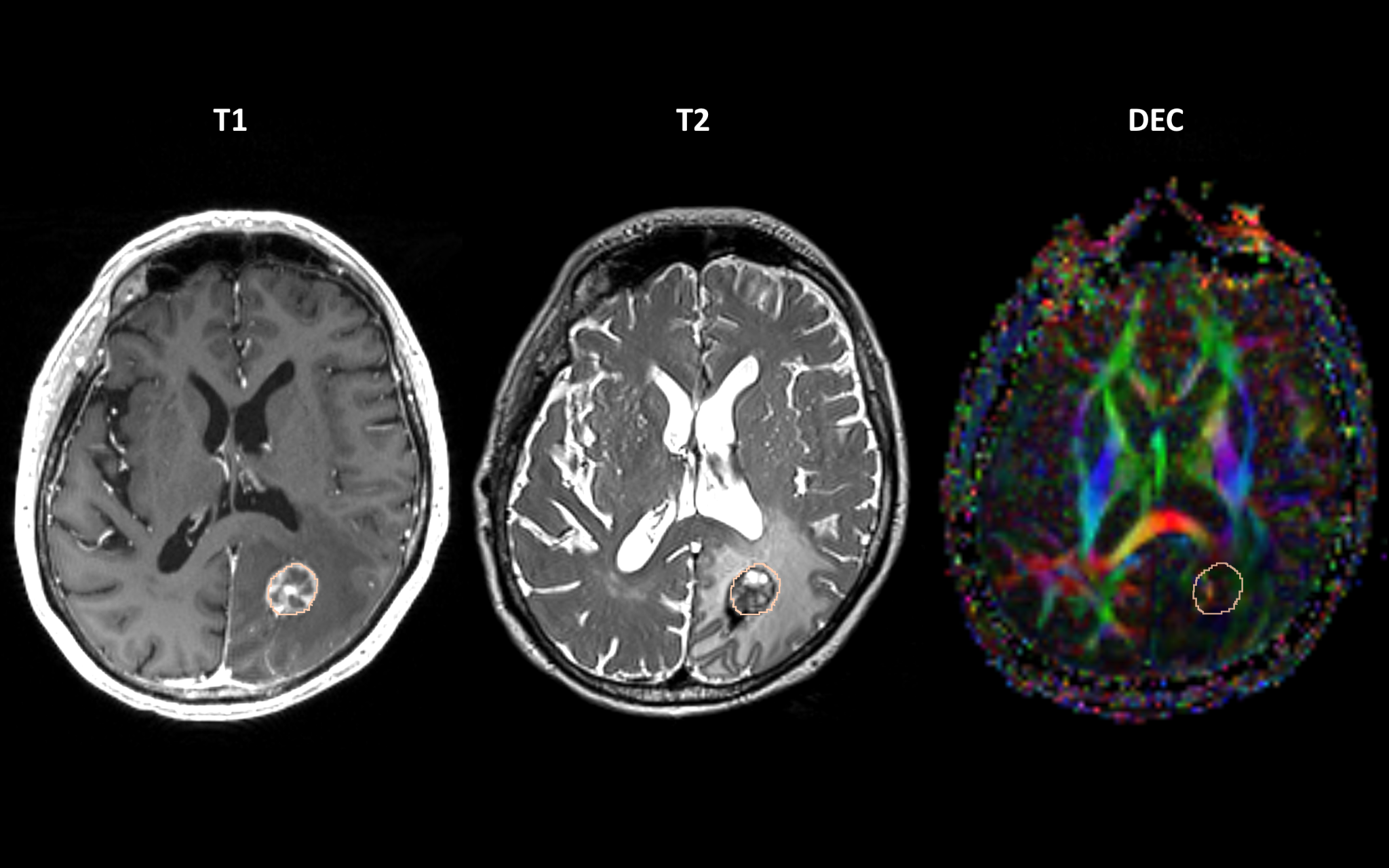
**

*BTP 1 DEC-TDI***
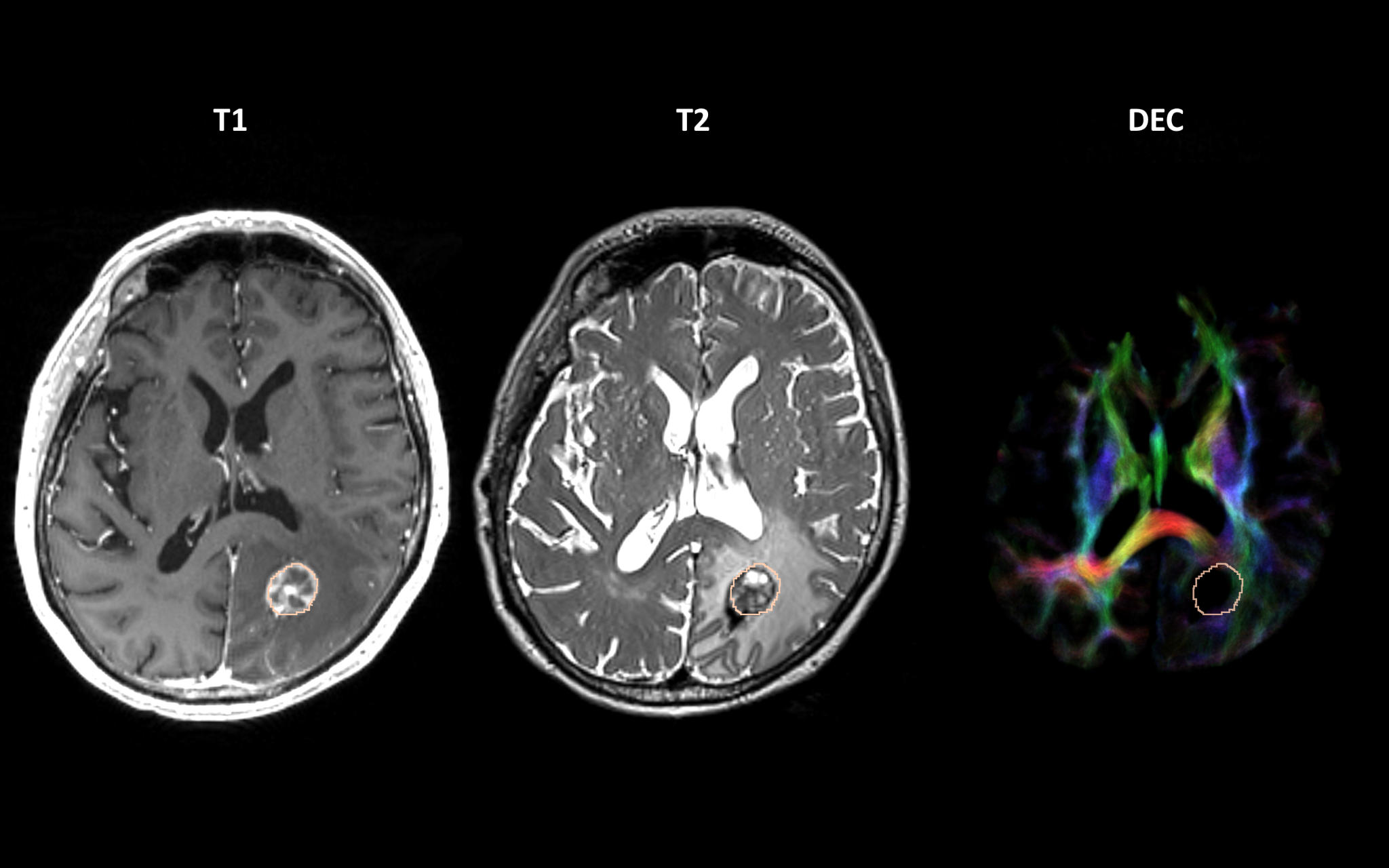
**

*BTP 2 DEC-FA*

**
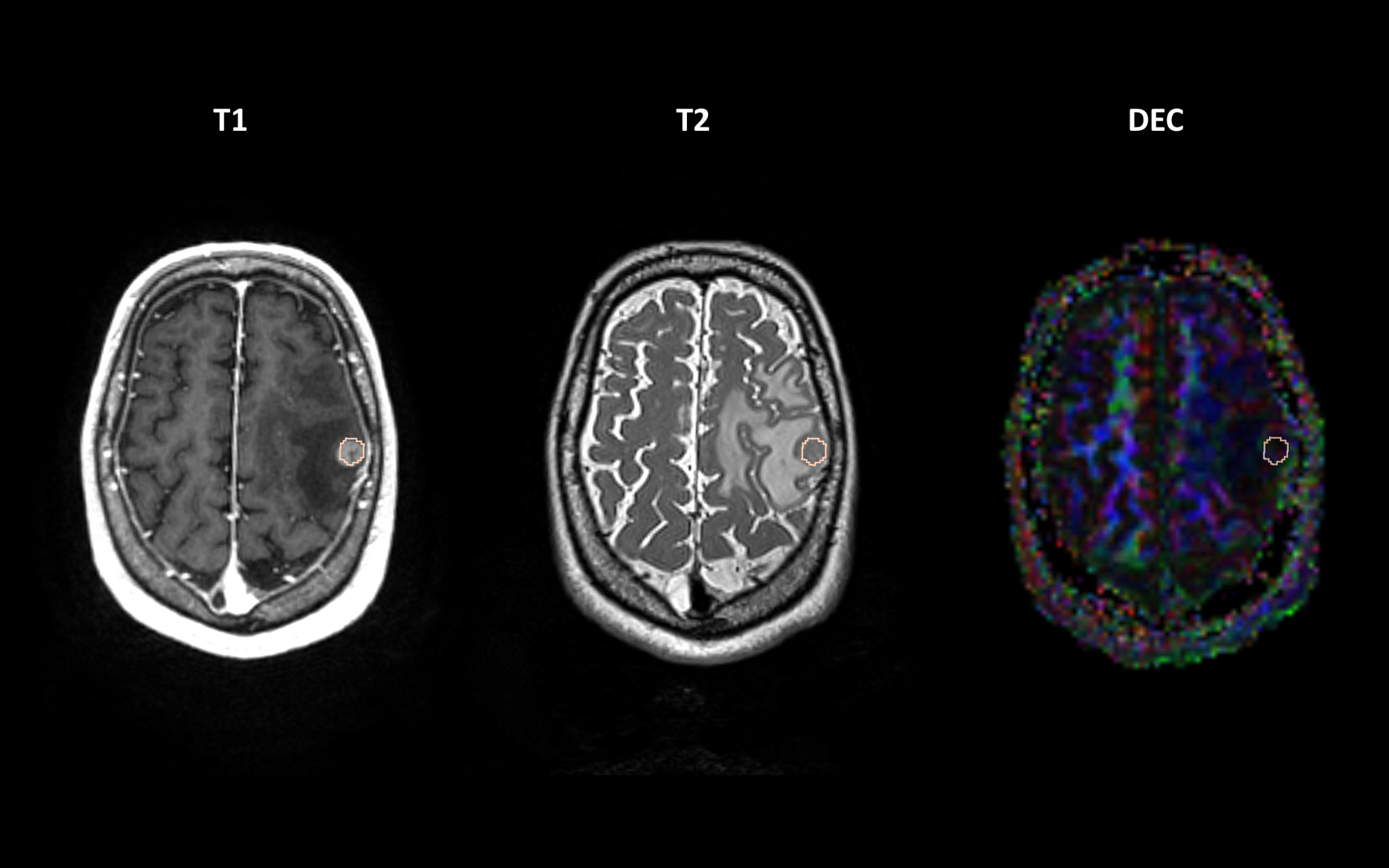
**

*BTP 2 DEC-TDI*

***
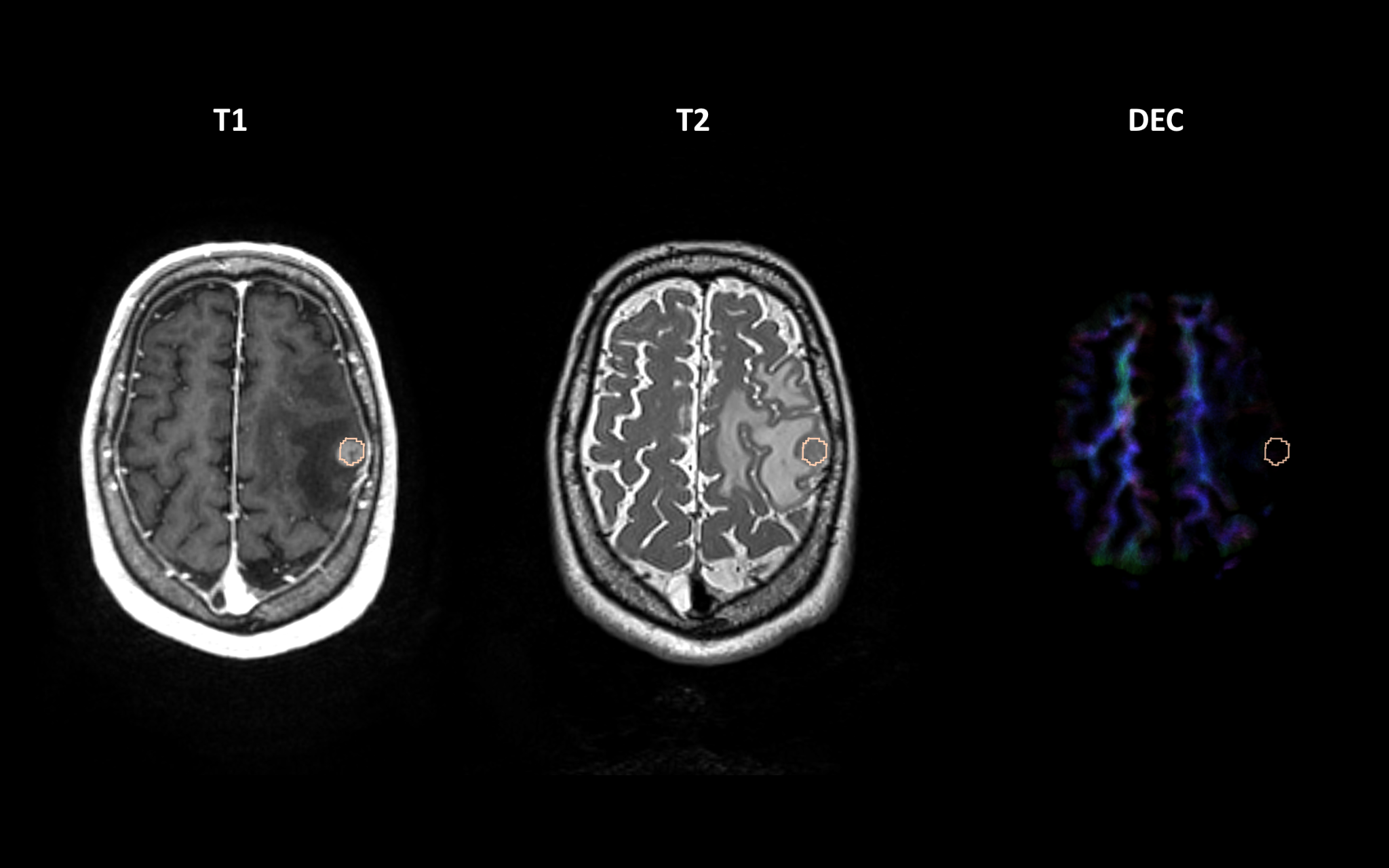
***

*BTP 3 DEC-FA*

*
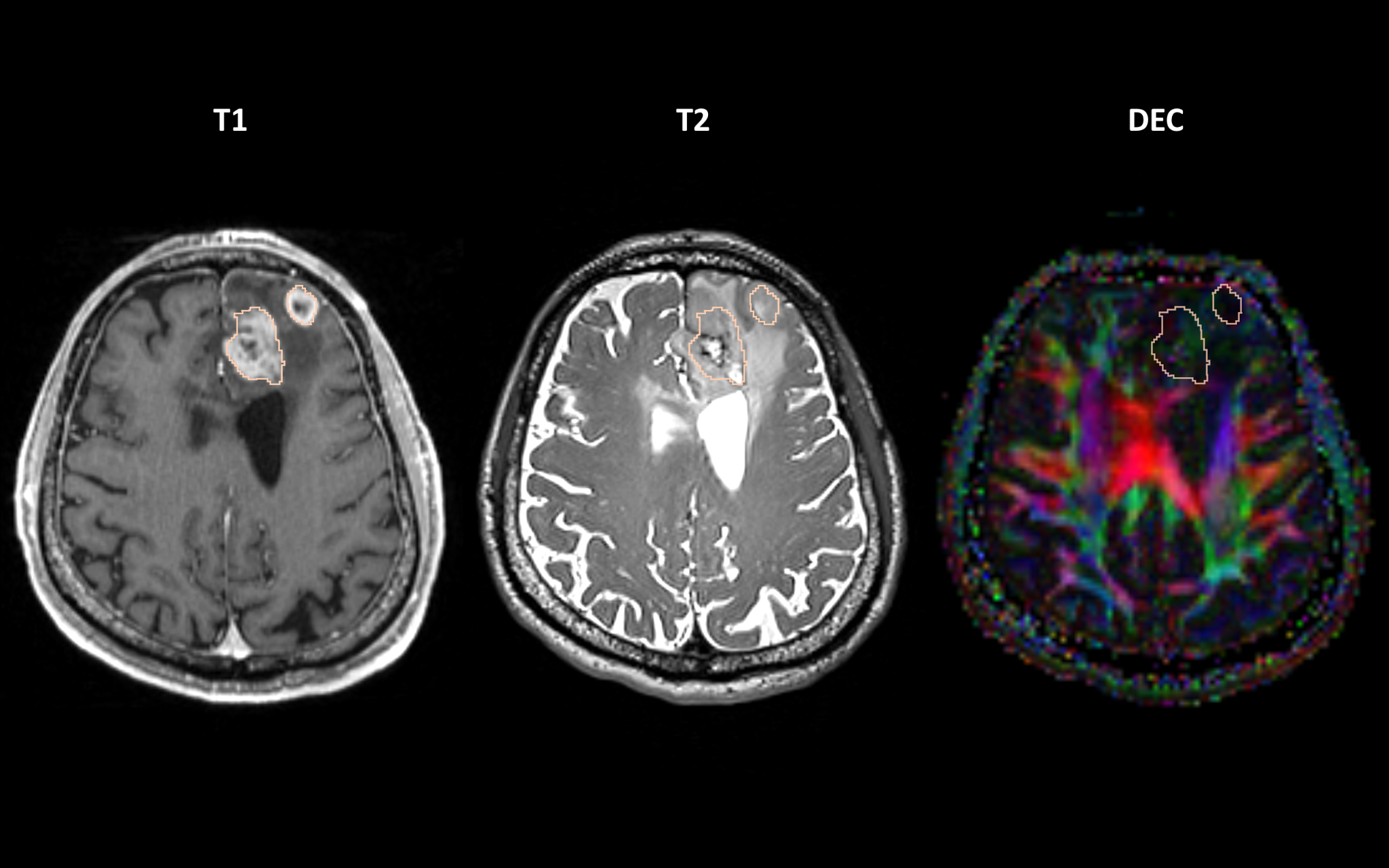
*

*BTP 3 DEC-TDI*

*
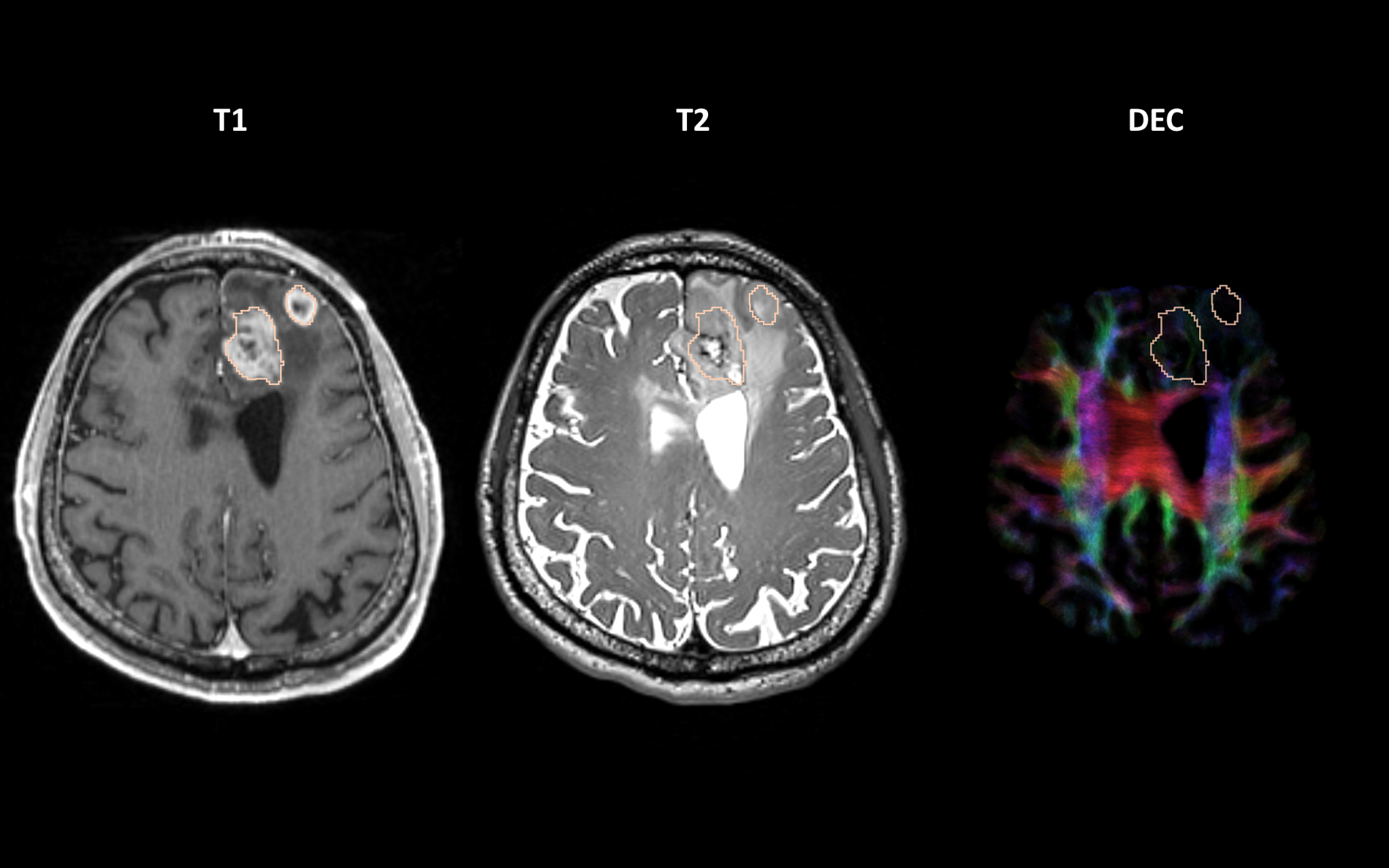
*

*BTP 4 DEC-FA*

*
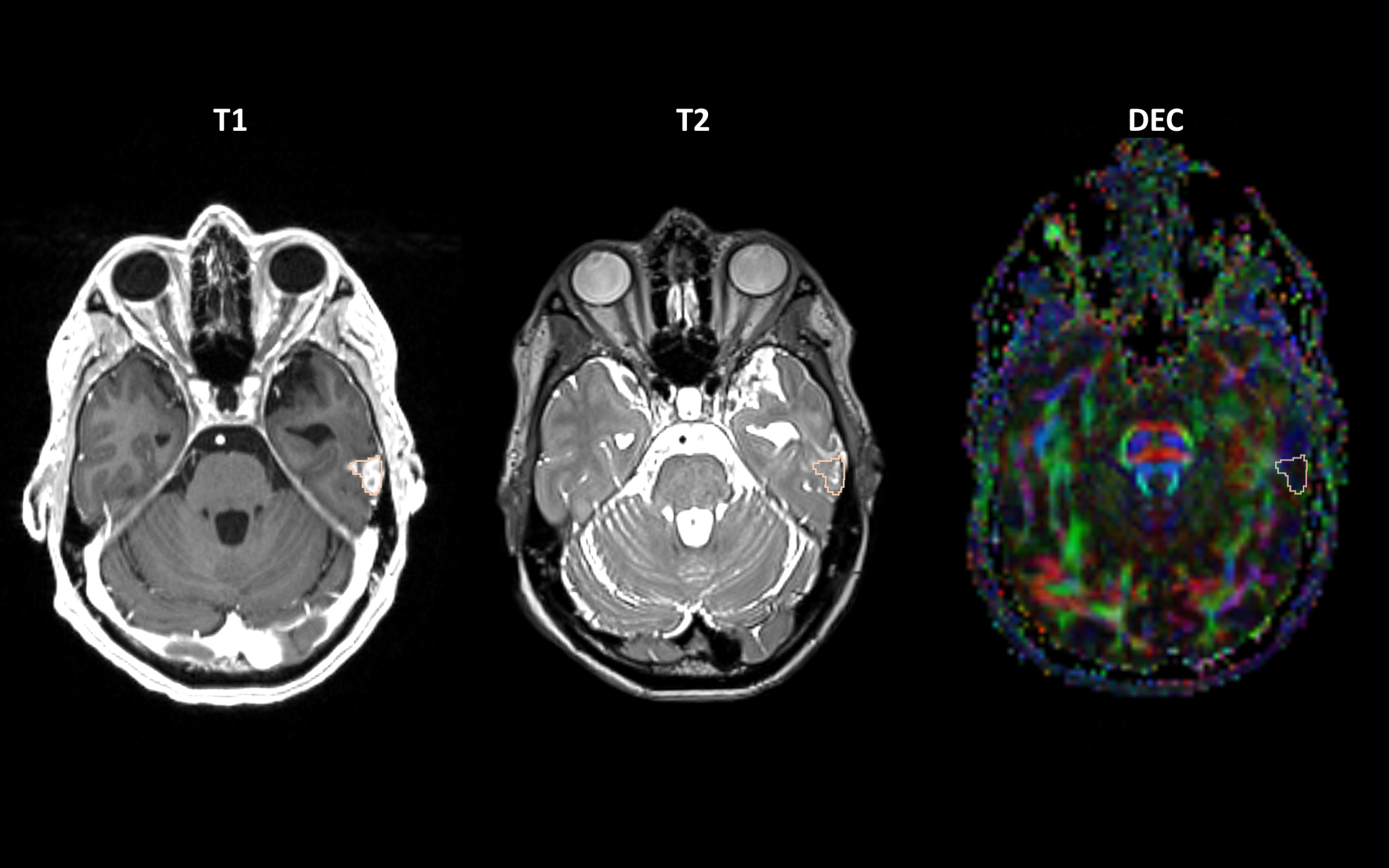
*

*BTP 4 DEC-TDI*

*
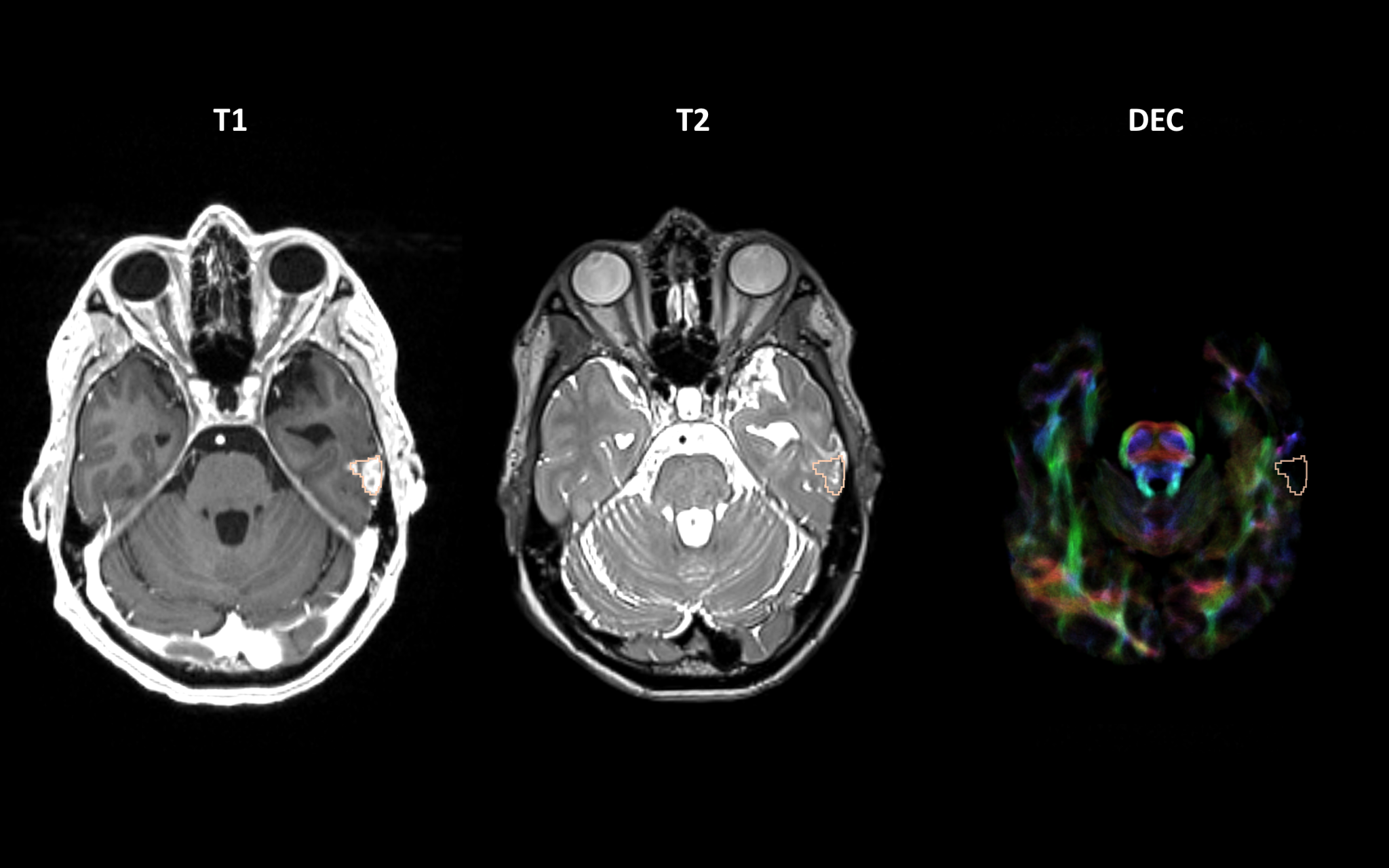
*

*BTP 5 DEC-FA*

*
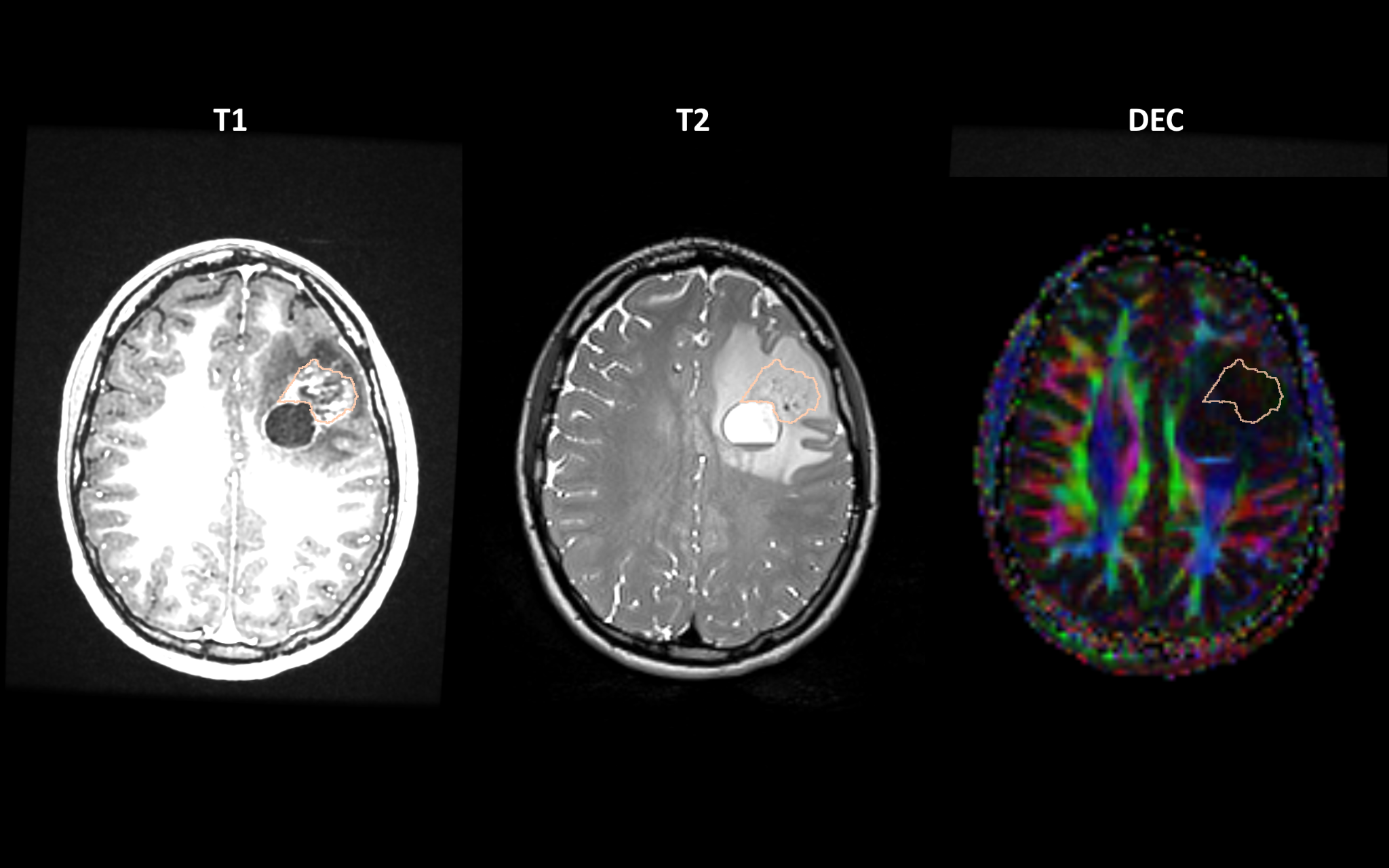
*

*BTP 5 DEC-TDI*

*
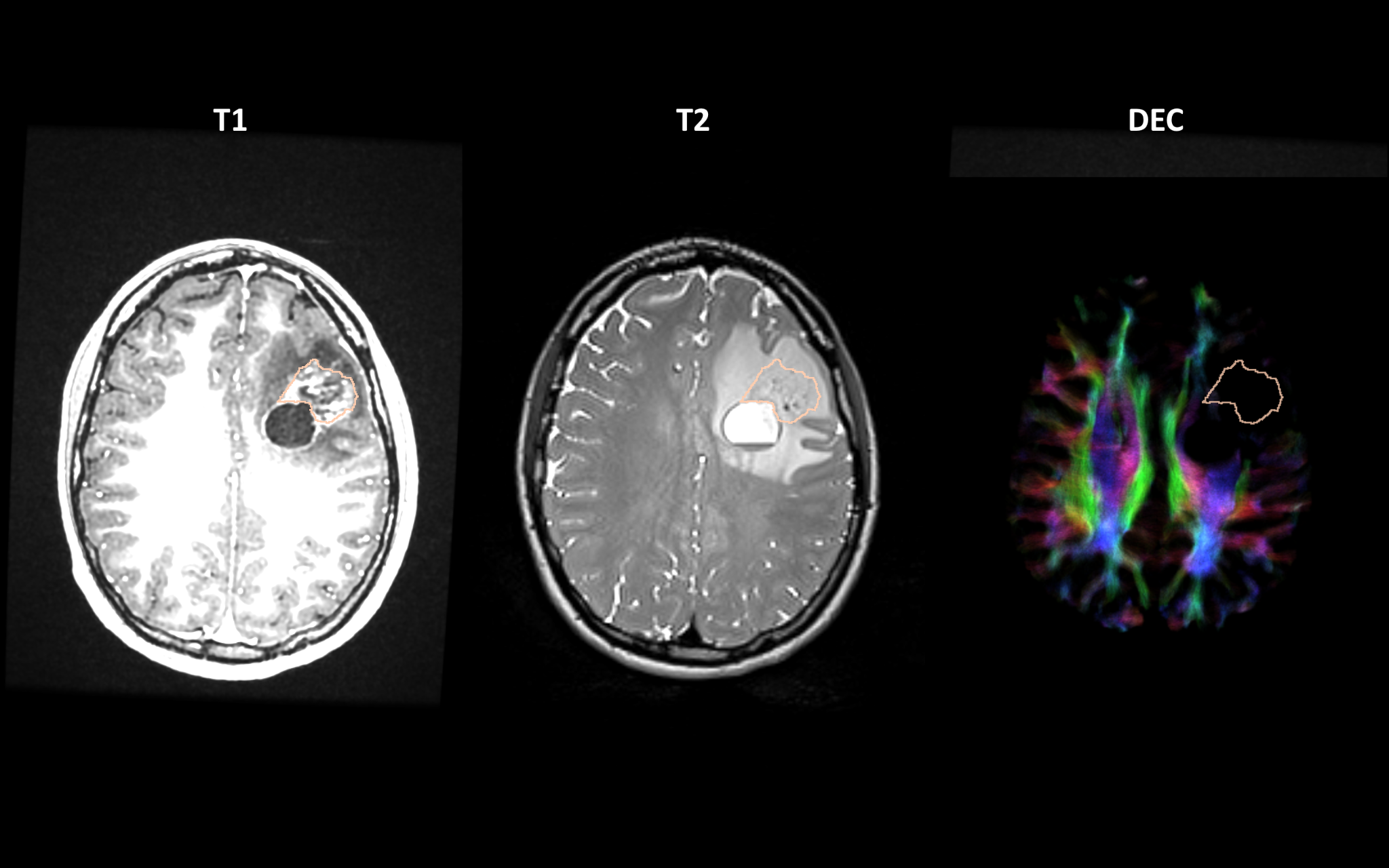
*

*BTP 6 DEC-FA*

*
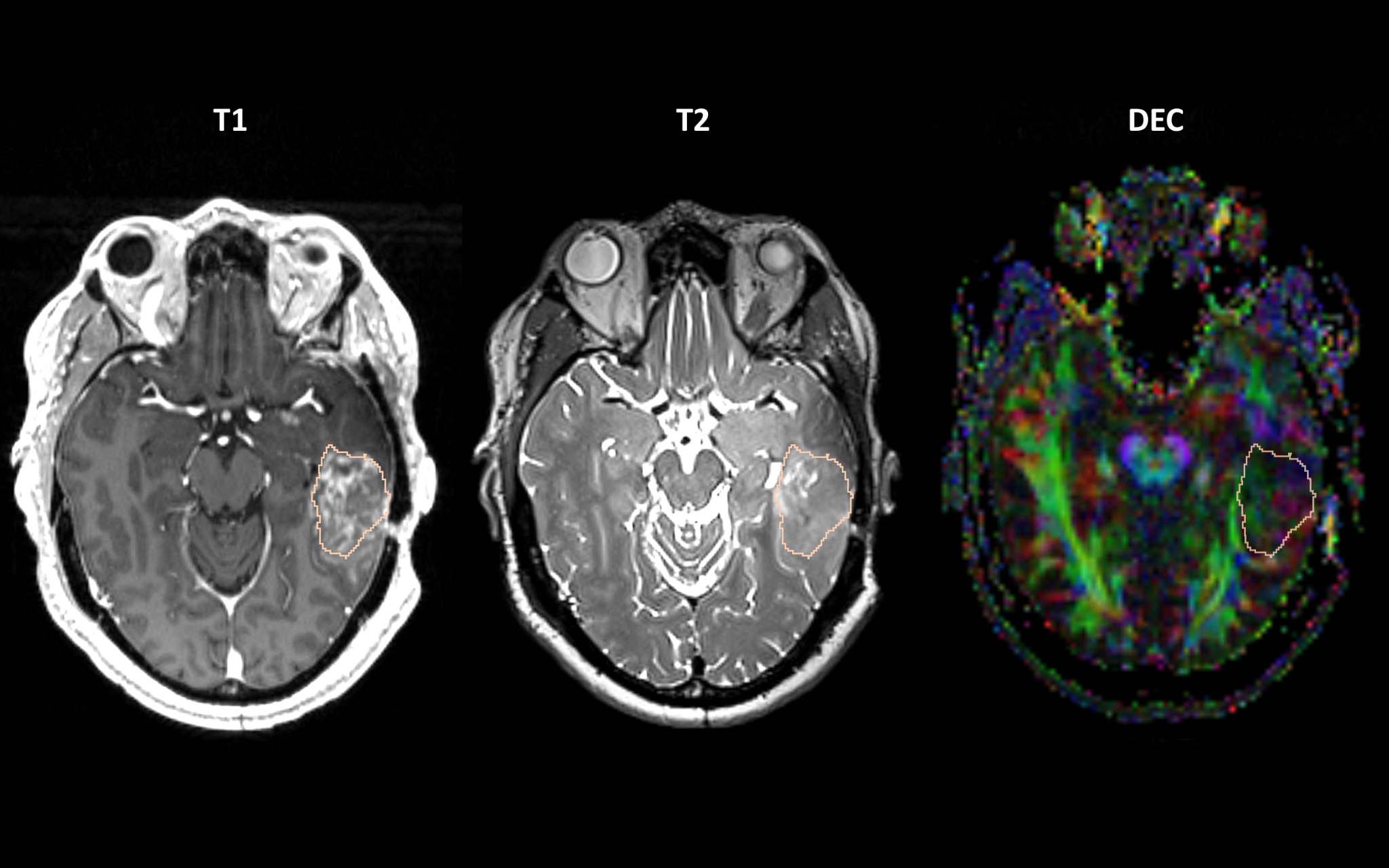
*

*BTP 6 DEC-TDI*

*
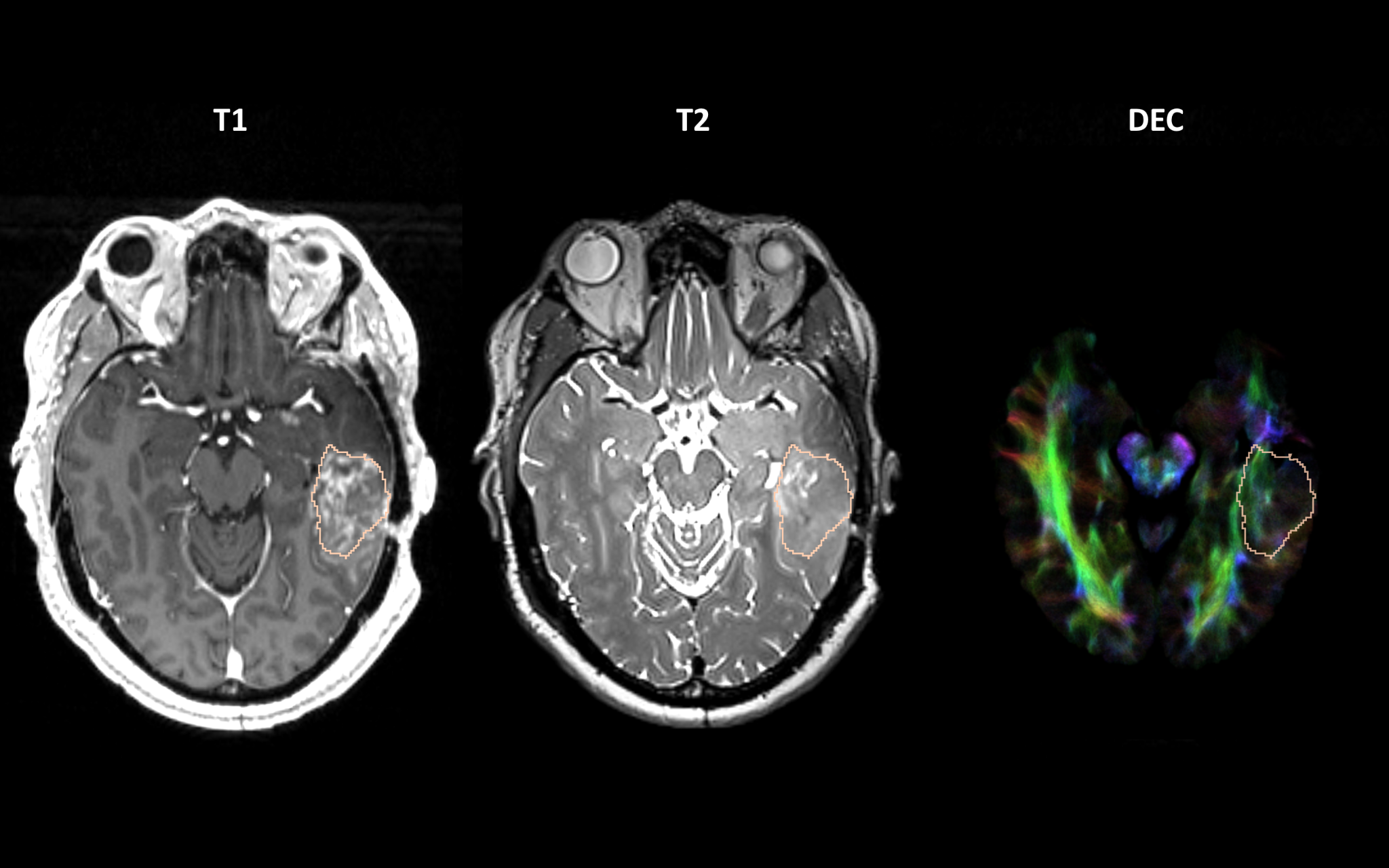
*

*BTP 7 DEC-FA*

*
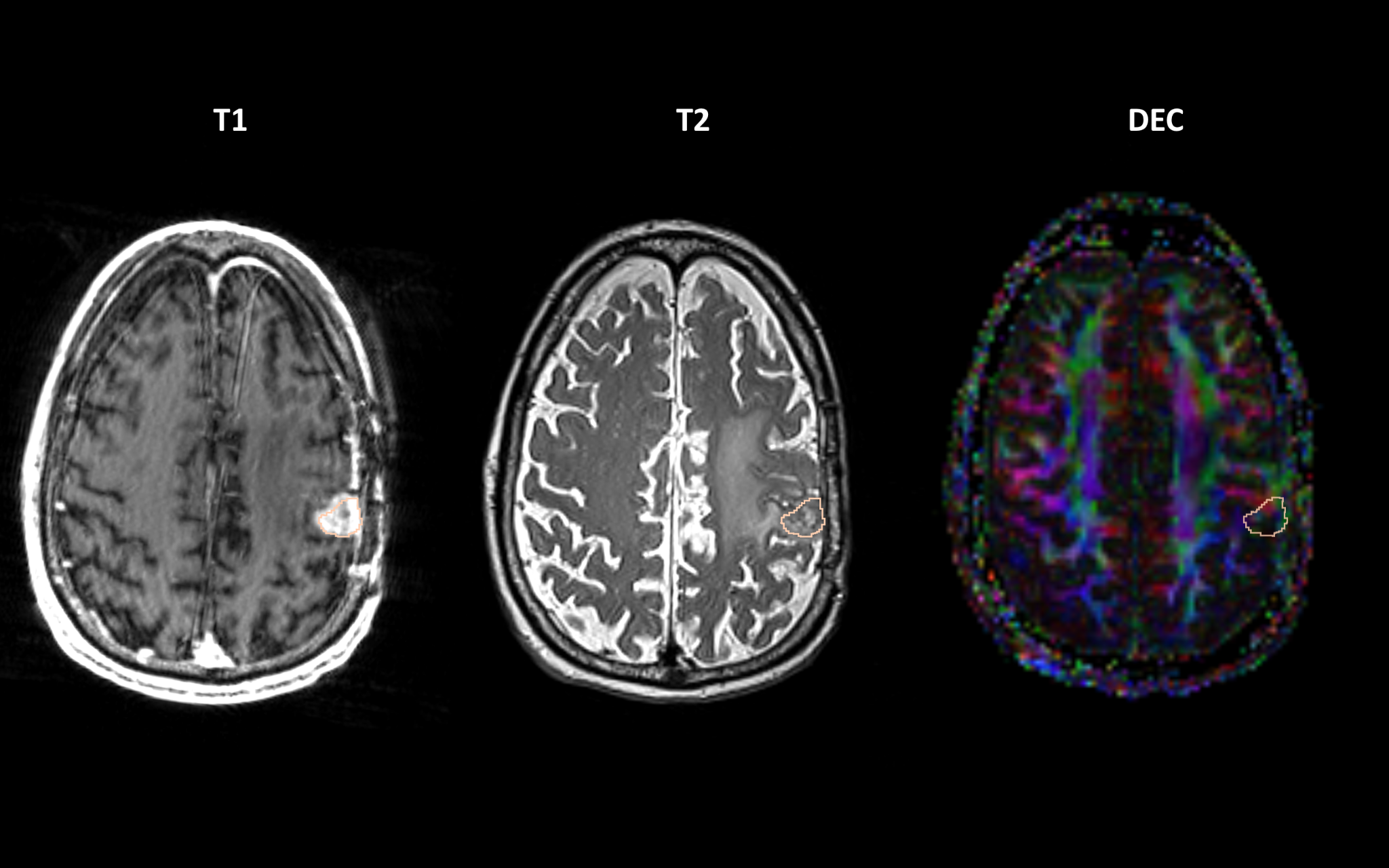
*

*BTP 7 DEC-TDI*

*
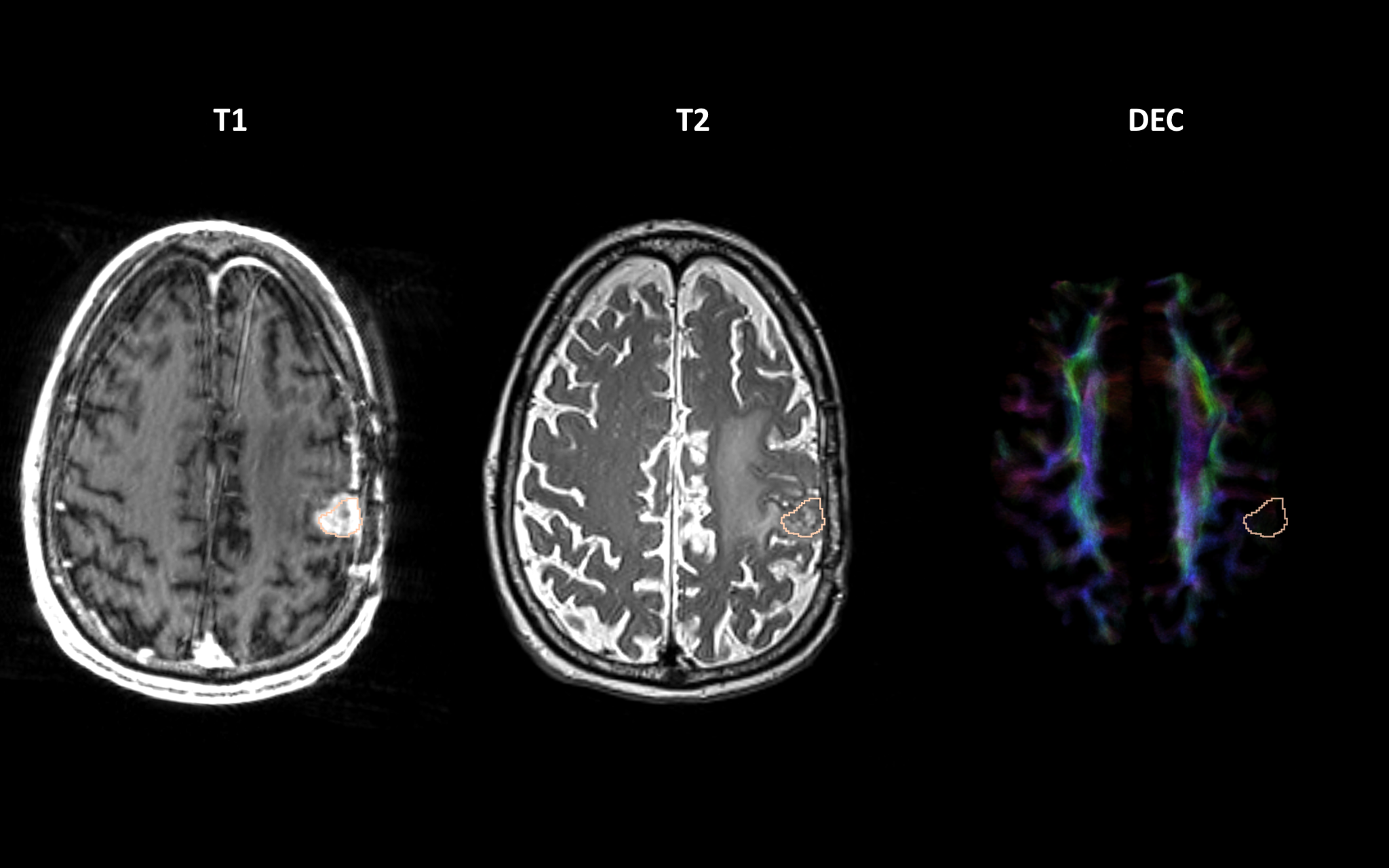
*

*BTP 8 DEC-FA*

*
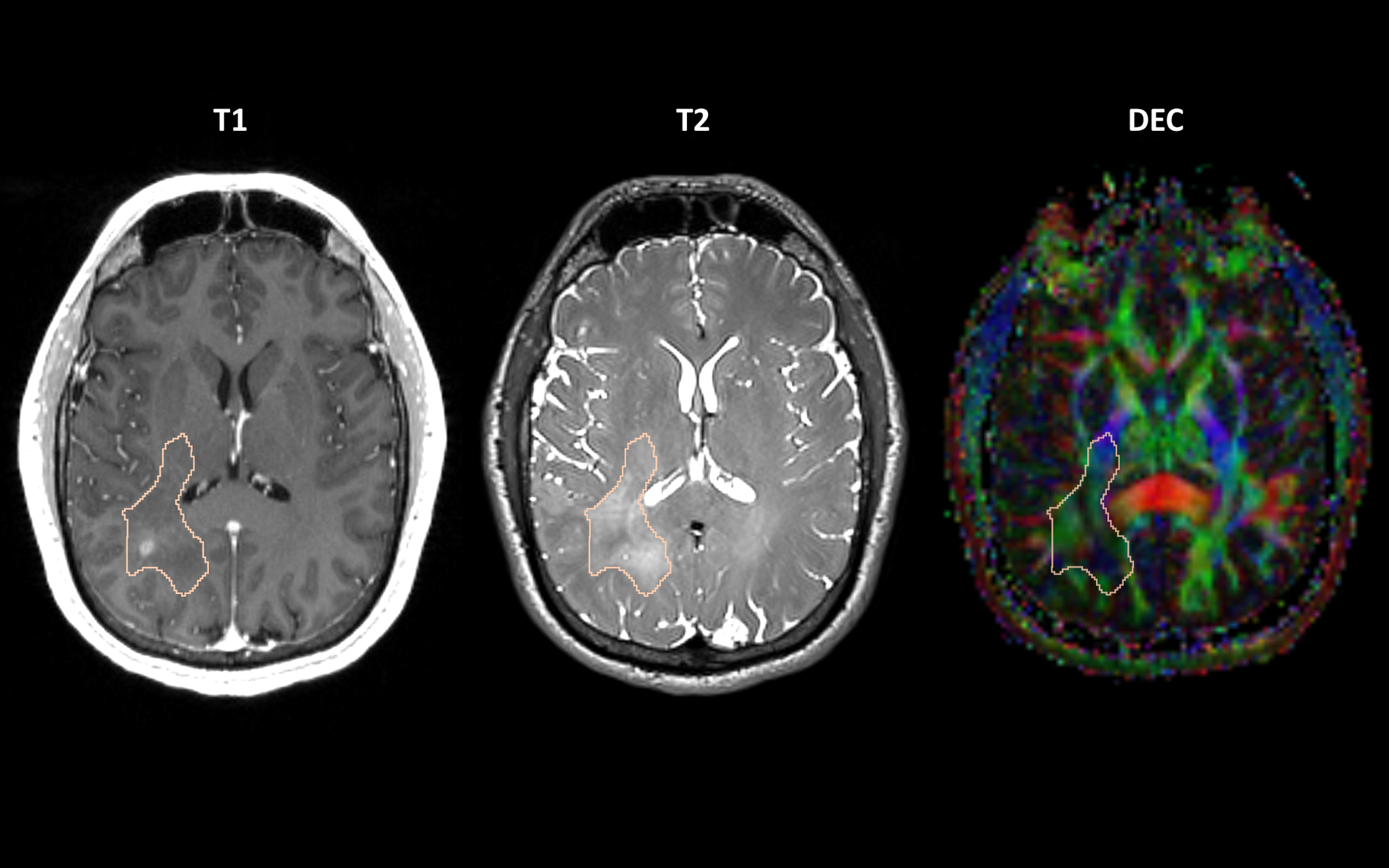
*

*BTP 8 DEC-TDI*

*
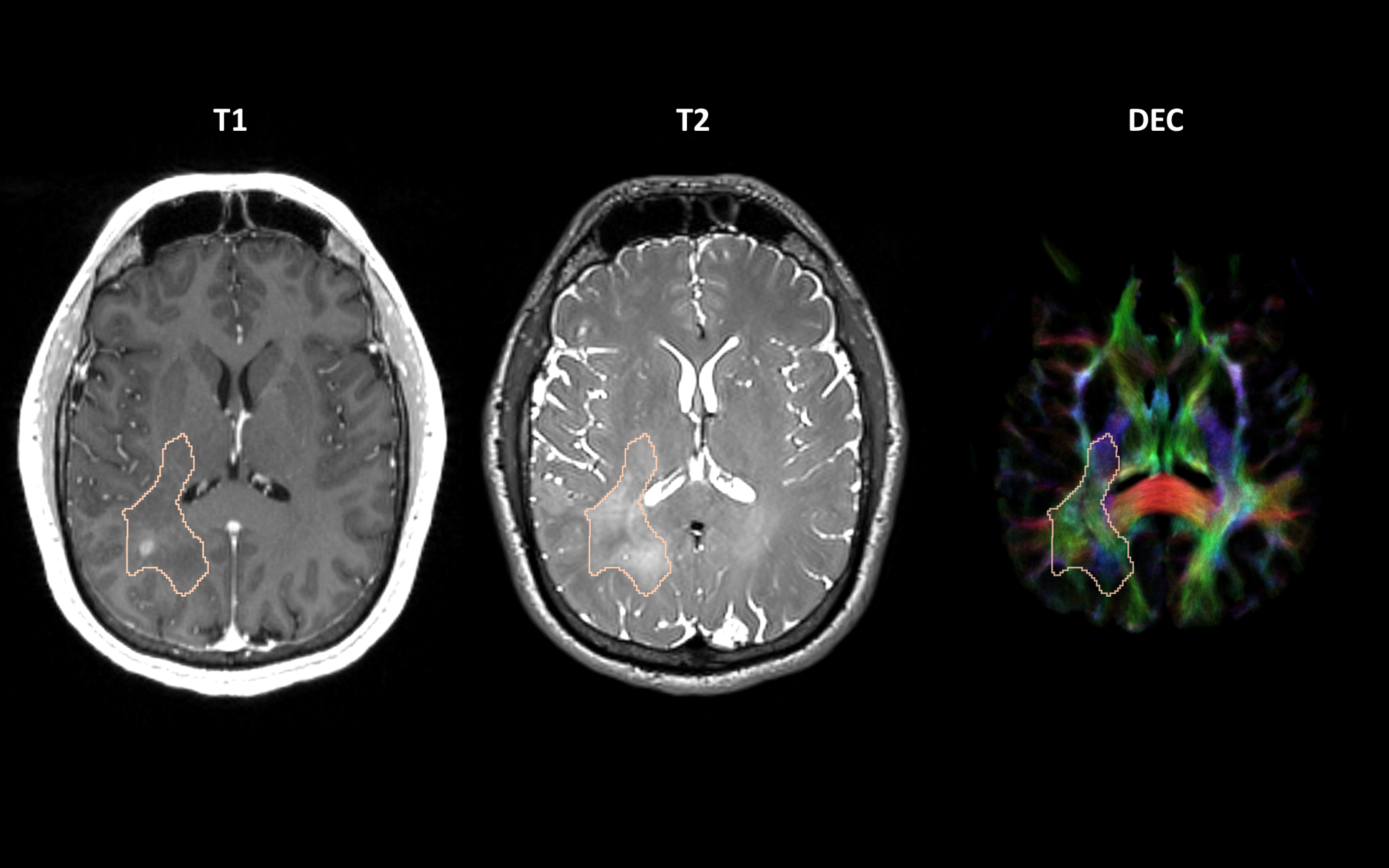
*

*BTP 9 DEC-FA*

*
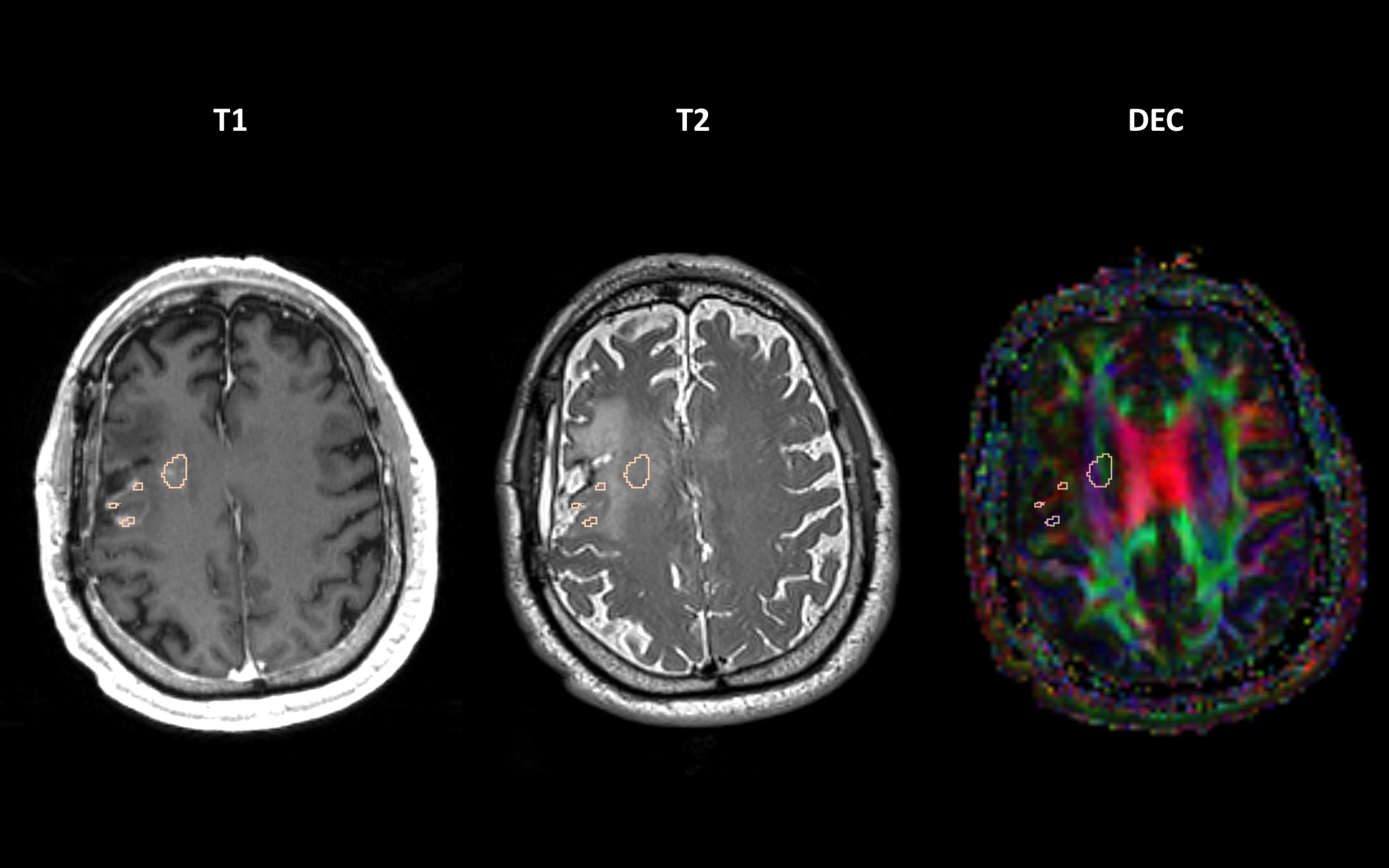
*

*BTP 9 DEC-TDI*

*
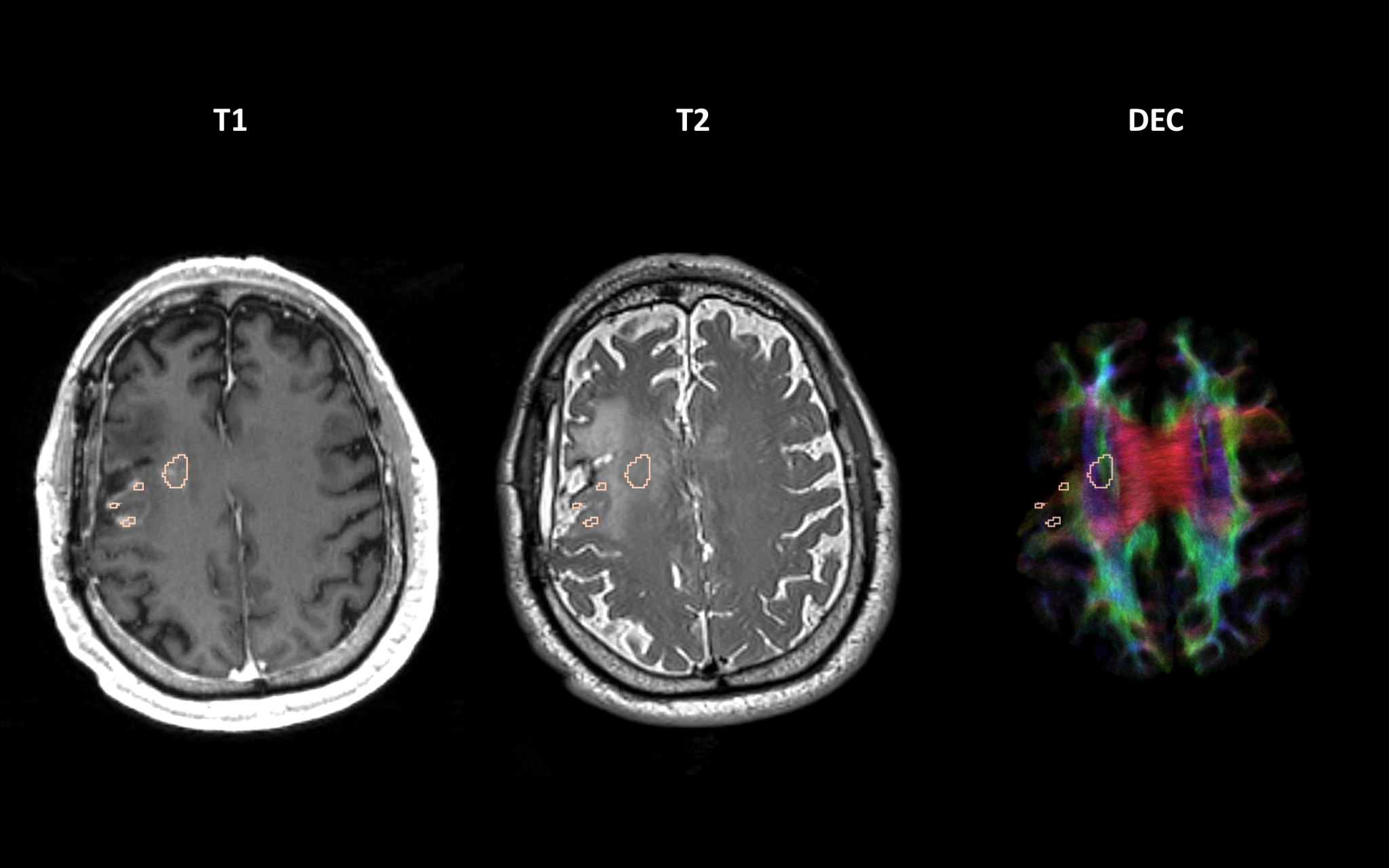
*

*BTP 10 DEC-FA*

*
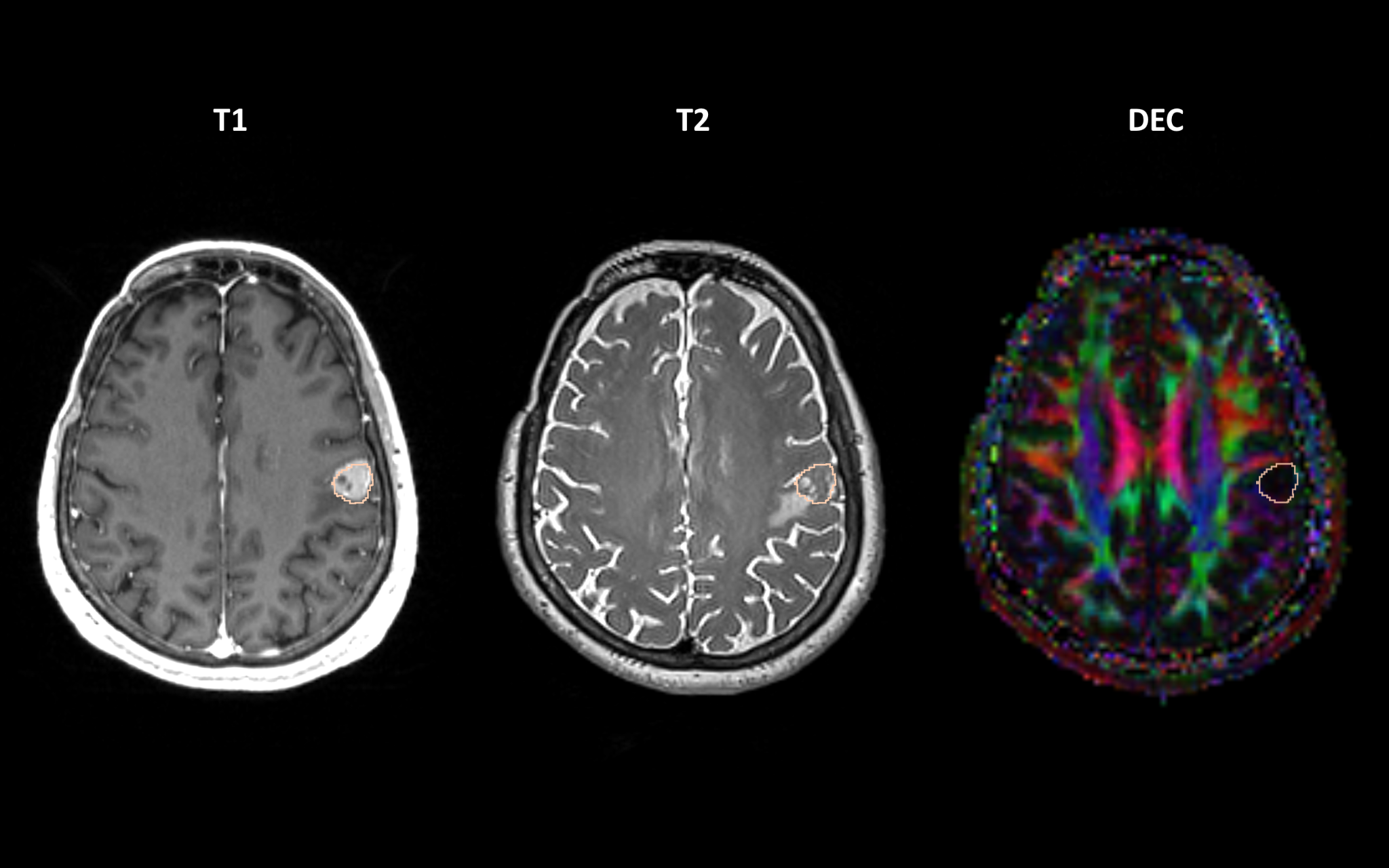
*

*BTP 10 DEC-TDI*

*
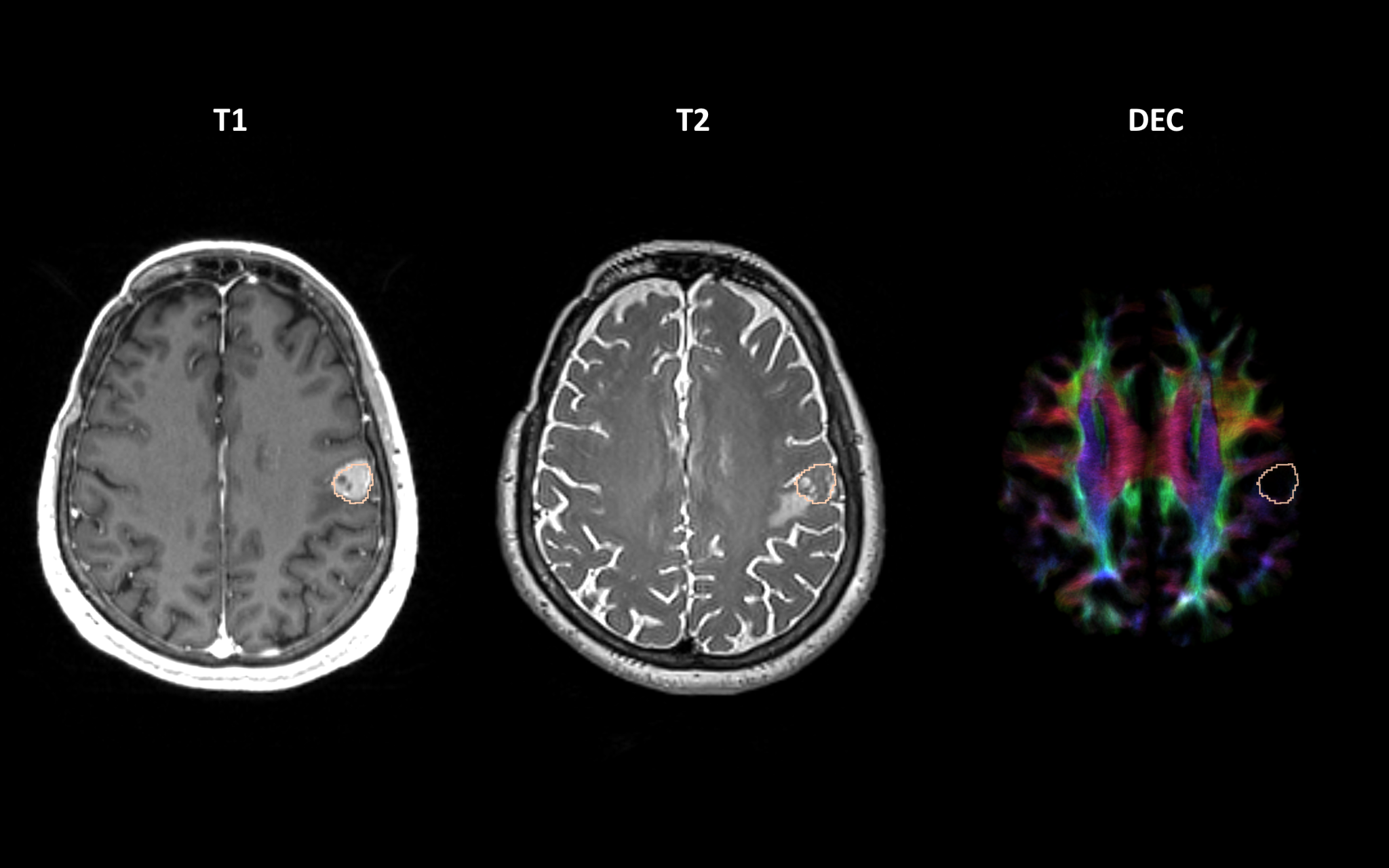
*

*BTP 11 DEC-FA*

*
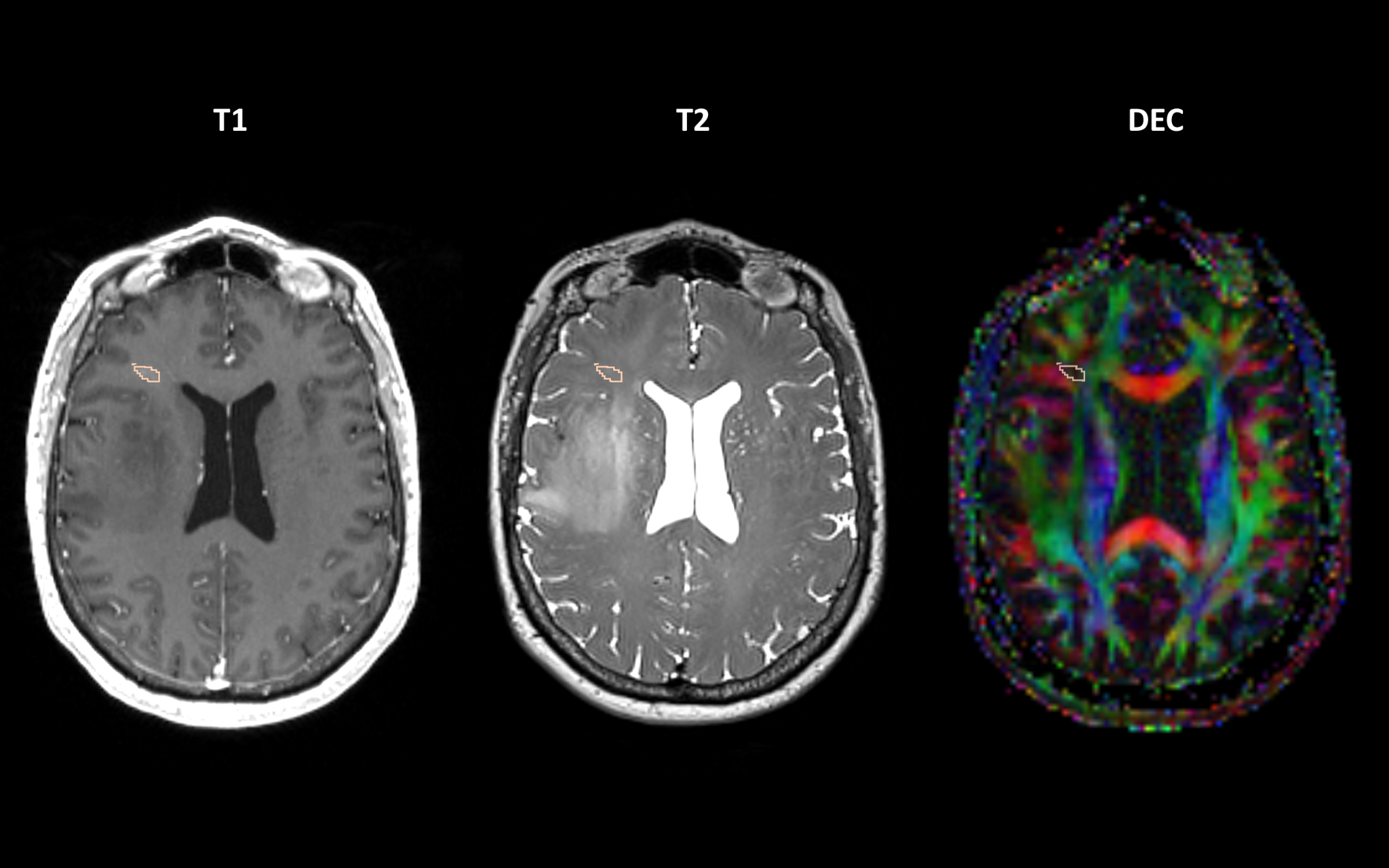
*

*BTP 11 DEC-TDI*

*
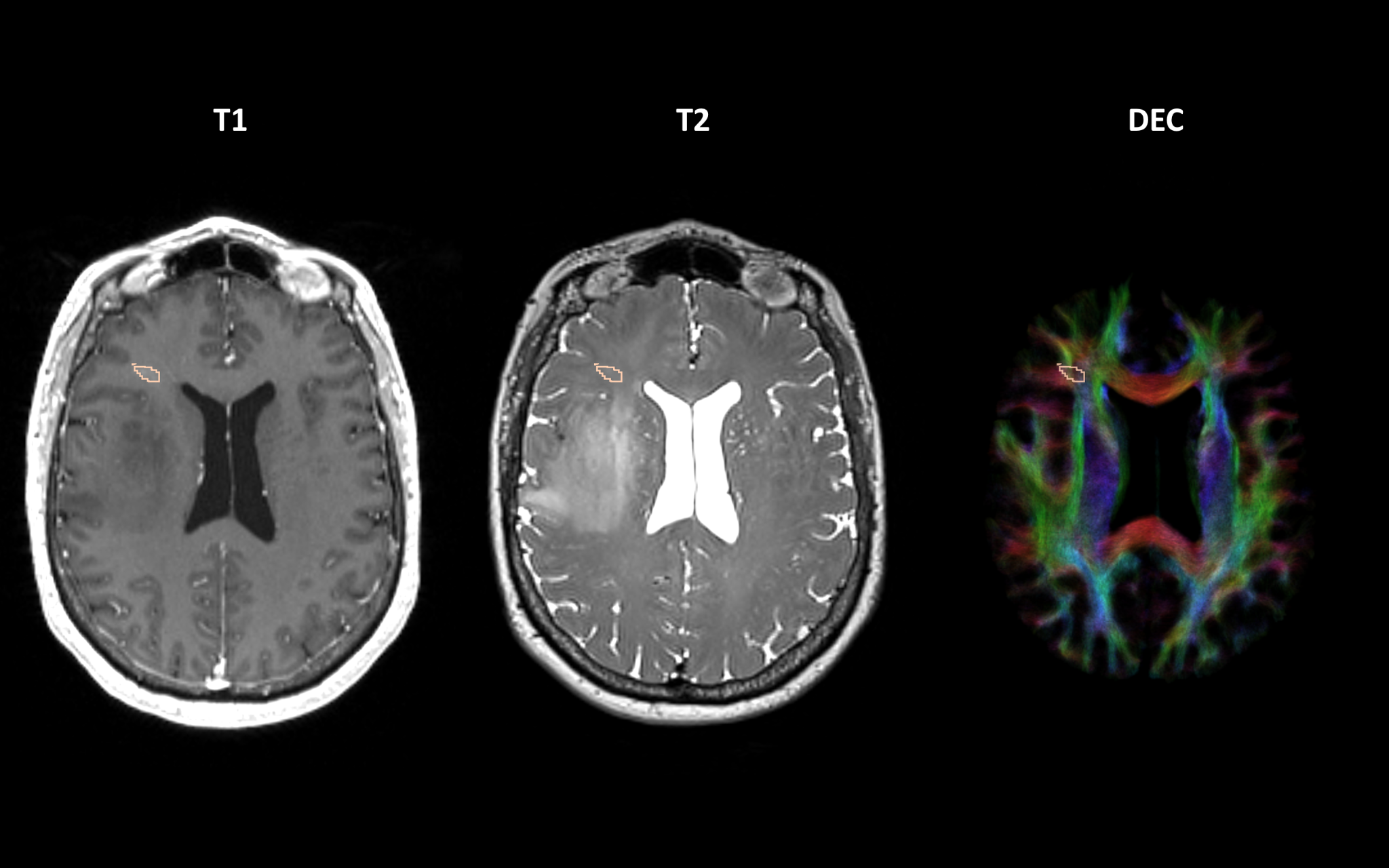
*

*BTP 12 DEC-FA*

*
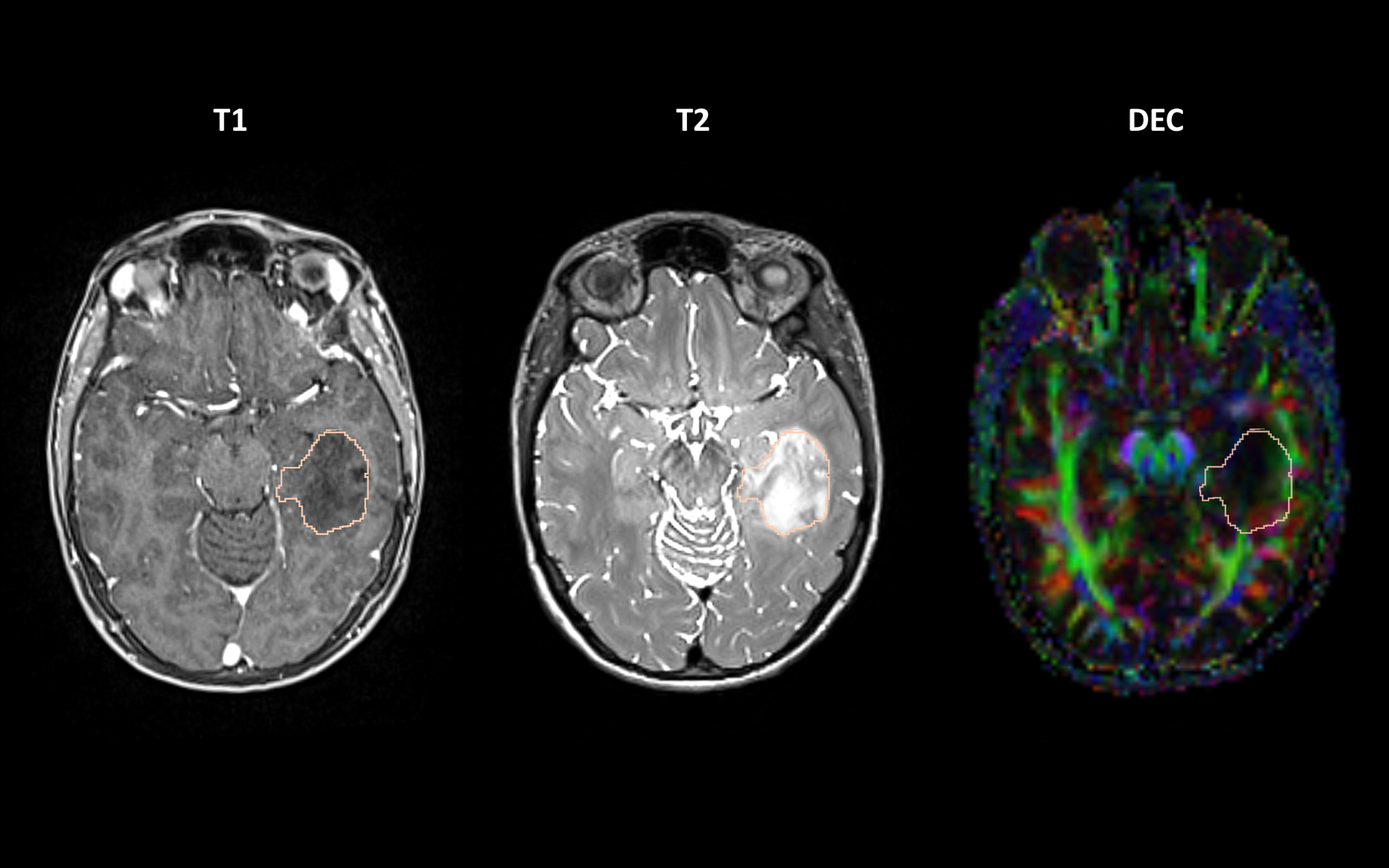
*

*BTP 12 DEC-TDI*

*
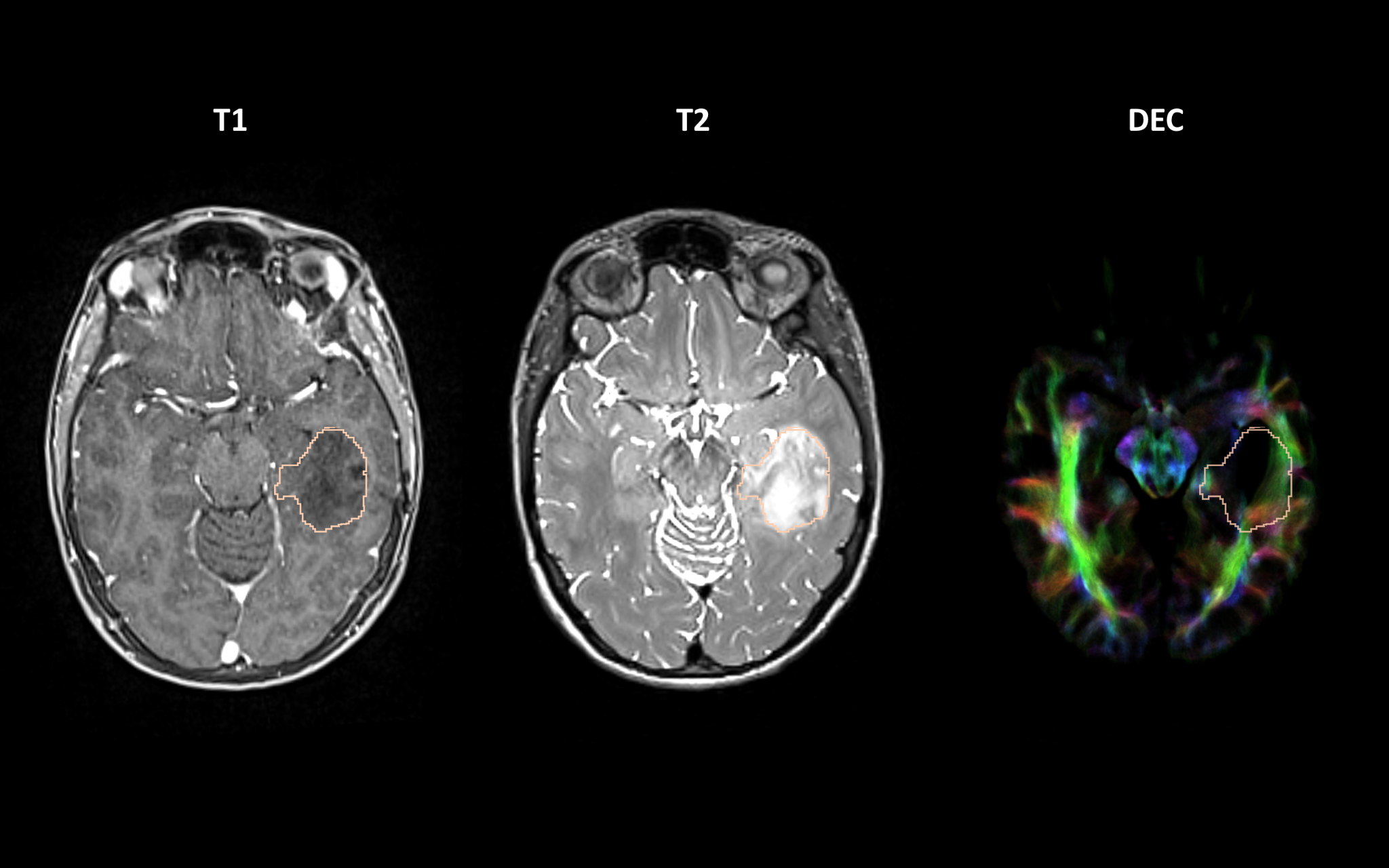
*

*BTP 13 DEC-FA*

*
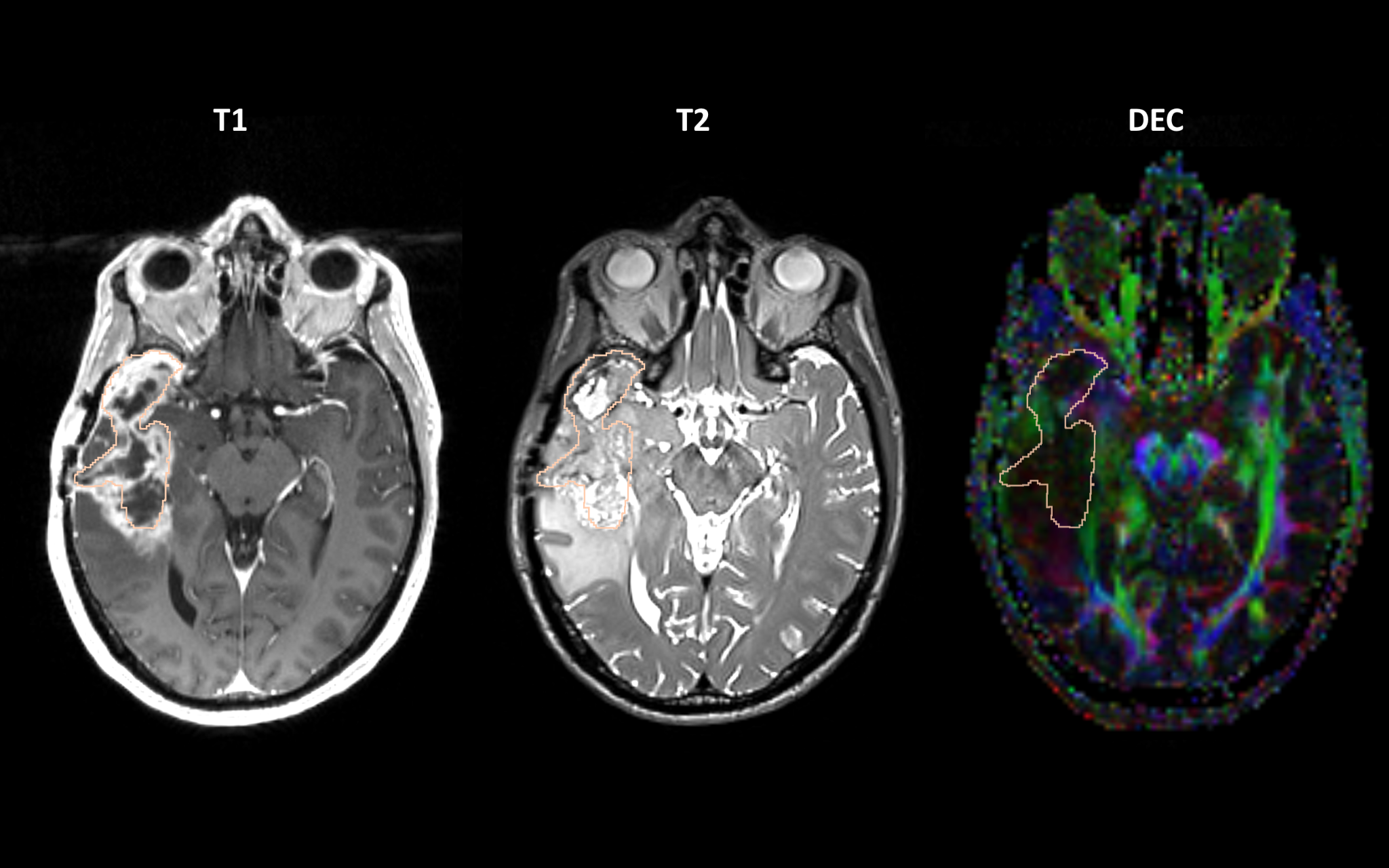
*

*BTP 13 DEC-TDI*

*
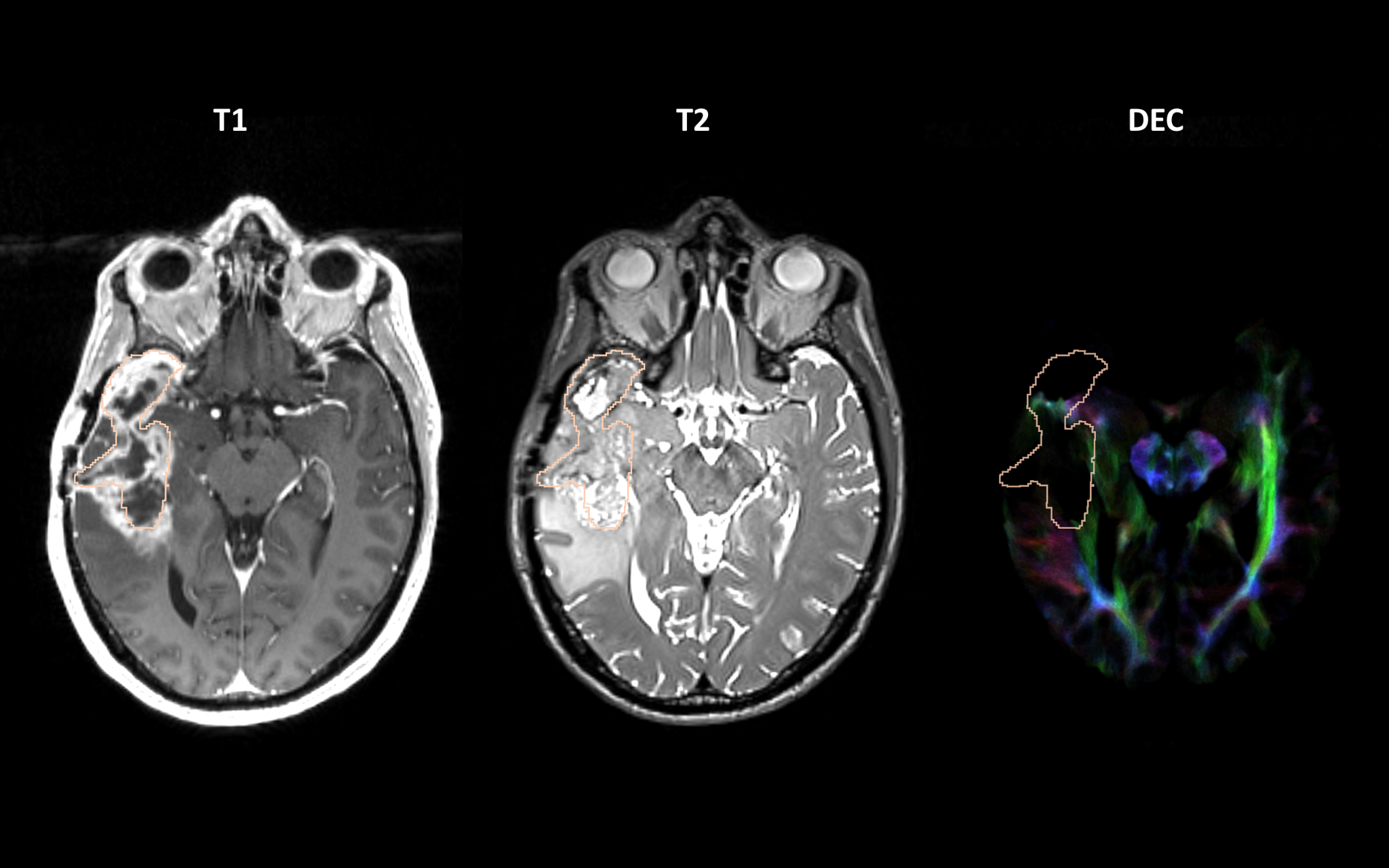
*

*BTP 14 DEC-FA*

*
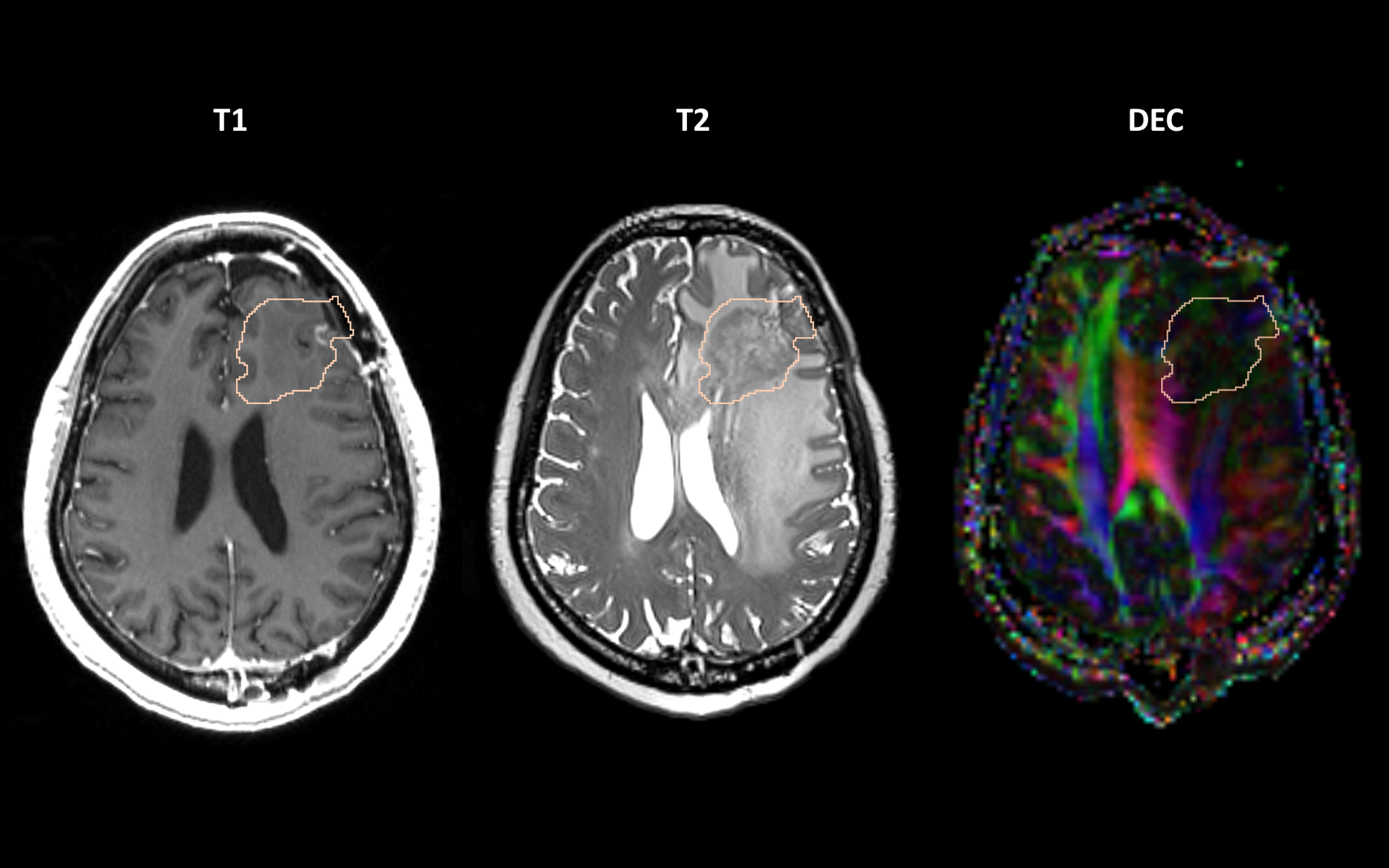
*

*BTP 14 DEC-TDI*

*
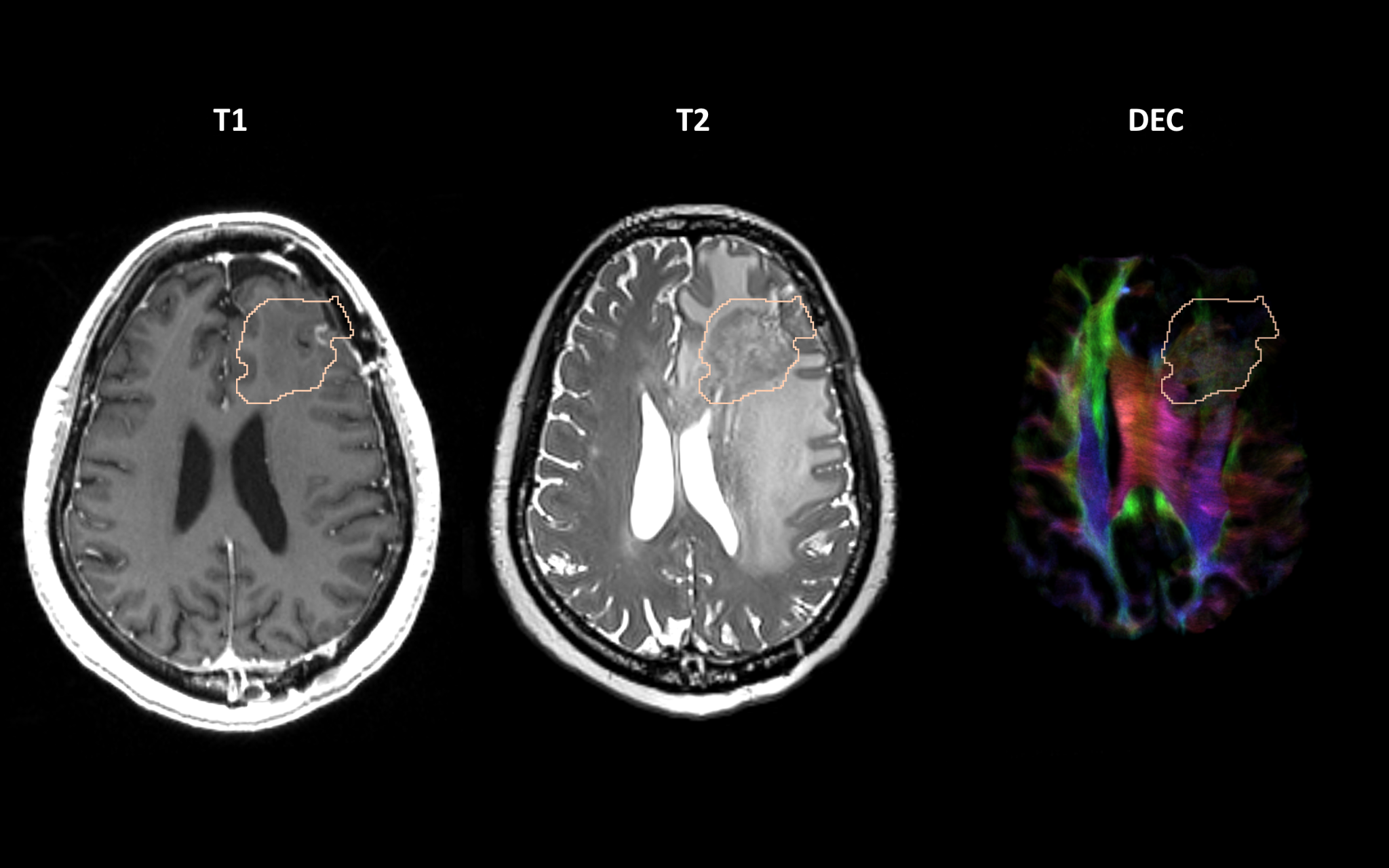
***Supplementary Fig. 1.** Visualization of all 28 DEC maps as presented to the raters. Displayed from left to right: T1-weighted image, T2-weighted image, DEC map. BTP = brain tumor patient, DEC = directionally encoded color, FA = fractional anisotropy, TDI = track density imaging.
